# Acquired Pedophilia: international Delphi-method-based consensus guidelines

**DOI:** 10.1038/s41398-023-02314-8

**Published:** 2023-01-18

**Authors:** Cristina Scarpazza, Cristiano Costa, Umberto Battaglia, Colleen Berryessa, Maria Lucia Bianchetti, Ilenia Caggiu, Orrin Devinsky, Stefano Ferracuti, Farah Focquaert, Arianna Forgione, Fredric Gilbert, Ambrogio Pennati, Pietro Pietrini, Innocenzo Rainero, Giuseppe Sartori, Russell Swerdlow, Andrea S. Camperio Ciani

**Affiliations:** 1 Department of General Psychology, University of Padova, Padova, Italy; 2 Padova Neuroscience Center (PNC), University of Padova, Padova, Italy; 3 IRCCS S. Camillo Hospital, Venezia, Italy; 4 Department of Neuroscience, University of Padova, Padova, Italy; 5 Department of Applied Psychology, FISPPA – University of Padova, Padova, Italy; 6 School of Criminal Justice, Rutgers University, Newark, NJ USA; 7 Epilepsy Center, NYU School of Medicine, New York, USA; 8 Department of Human Neurosciences Sapienza Università di Roma, Rome, Italy; 9 Bioethics Institute Ghent, Department of Philosophy and Moral Sciences, Ghent University, Ghent, Belgium; 10 Ethics, Policy & Public Engagement (EPPE) ARC Centre of Excellence for Electromaterials Science (ACES), Faculty of Arts, University of Tasmania, Hobart, Australia; 11 Associazione Scientifica Integrational Mind Labs, Milano, Italy; 12 IMT School for Advanced Studies Lucca, Lucca, Italy; 13 Neurology I, Department of Neuroscience “Rita Levi Montalcini”, University of Torino, Turin, Italy; 14 University of Kansas Medical Center, Kansas City, KS USA

**Keywords:** Psychiatric disorders, Neuroscience

## Abstract

Idiopathic and acquired pedophilia are two different disorders with two different etiologies. However, the differential diagnosis is still very difficult, as the behavioral indicators used to discriminate the two forms of pedophilia are underexplored, and clinicians are still devoid of clear guidelines describing the clinical and neuroscientific investigations suggested to help them with this difficult task. Furthermore, the consequences of misdiagnosis are not known, and a consensus regarding the legal consequences for the two kinds of offenders is still lacking. The present study used the Delphi method to reach a global consensus on the following six topics: behavioral indicators/red flags helpful for differential diagnosis; neurological conditions potentially leading to acquired pedophilia; neuroscientific investigations important for a correct understanding of the case; consequences of misdiagnosis; legal consequences; and issues and future perspectives. An international and multidisciplinary board of scientists and clinicians took part in the consensus statements as Delphi members. The Delphi panel comprised 52 raters with interdisciplinary competencies, including neurologists, psychiatrists, neuropsychologists, forensic psychologists, expert in ethics, etc. The final recommendations consisted of 63 statements covering the six different topics. The current study is the first expert consensus on a delicate topic such as pedophilia. Important exploitable consensual recommendations that can ultimately be of immediate use by clinicians to help with differential diagnosis and plan and guide therapeutic interventions are described, as well as future perspectives for researchers.

## Introduction

Pedophilia is a disorder of public concern because of its association with child sexual offense and recidivism [1]. The fifth edition of the Diagnostic and Statistical Manual of Psychiatric Disorder (DSM5) clearly distinguishes between pedophilia and pedophilic behavior, defining pedophilia as a sexual attraction toward children, while stating that pedophilic behavior can be diagnosed only when pedophilic urges cause distress in the individual with pedophilia or result in sexual offenses toward children [2, 3].

The literature clearly states that many psychiatric symptoms can be indicative of an underlying neurological disorder (i.e., alcoholic hallucinosis [4], secondary psychoses [5]) [6, 7]. For this reason, for most of the psychiatric conditions, the DSM5 includes a diagnostic criterion stating that psychiatric diagnosis can only be made if *“the symptoms are not attributable to the physiological effects of a substance or another medical conditions”* [2]. This diagnostic criterion is not present for paraphilias and, in particular, pedophilia, even if pedophilic tendencies and behaviors might, in rare cases, have a neurologic basis [8].

Acquired pedophilic behavior (hereafter referred to as acquired pedophilia for brevity) refers to the insurgence of enacted pedophilic interests and/or urges as a symptom of brain insult of neurological origin [9, 10]. The onset of acquired pedophilia has been described as a consequence of frontotemporal dementia [11, 12], traumatic brain injury [13], surgical lesion [14], brain [15], or notochord [16] neoplasms, and multiple sclerosis [17, 18] in individuals who have never overtly manifested pedophilic behavior before.

For this reason, acquired pedophilia is different from developmental (or idiopathic) pedophilia, which, in contrast, is a psychiatric condition included within the section of paraphilias within the DSM5 [2].

Differences between psychiatric and neurologic disorders are pivotal to understanding the differences between the two forms of pedophilia.

Most likely, the most important difference between the two forms of brain disorders is the presence of biomarkers; this is a characteristic that can be objectively measured and evaluated as an indicator of normal biological processes, pathogenic processes, or pharmacologic responses to a therapeutic intervention [19]. Critically, while biomarkers are available for neurological disorders, thus supporting clinicians in the diagnostic process, they are still unavailable for psychiatric disorders [20, 21]. For instance, atrophy of the hippocampus and mesial temporal structures is a well-known biomarker for Alzheimer’s disease [22], as well as increased tau and decreased amyloid protein levels in the cerebrospinal fluid [23]; furthermore, biomarkers are included within the supporting diagnostic criteria [22]. The presence of oligoclonal bands is a biomarker for multiple sclerosis, and the performance of a spinal tap is thus indispensable to formulate this diagnosis [24]. Nothing similar is available for psychiatric disorders; there are no blood tests or other scientific exams that can help clinicians in the difficult task of psychiatric diagnosis. Indeed, the few proposed potential biomarkers, such as larger ventricles or reduced total gray matter in schizophrenia [25], suffer from limited accuracy and generalizability to real-life clinical settings [26, 27].

While neurological disorders are usually characterized by a clear neural basis, which is evident by magnetic resonance imaging (MRI) or positron emission tomography (PET), the brains of patients suffering from psychiatric disorders are *prima facie* indistinguishable from the brains of individuals free of such pathologies [28, 29]. In the last few decades, sophisticated brain imaging analysis has enriched the literature supporting the evidence of subtle brain alterations in patients with psychiatric disorders [28, 30–33]. However, these alterations, contrary to the neurological ones, are (i) not visible to the naked eye; (ii) identifiable only after complex statistical analysis of the brain; (iii) not consistent across different studies; and (iv) often unreliable at the level of the single individual. For this reason, the algorithms and online tools that have been created to support clinicians in the identification and quantification of brain abnormalities cannot be applied to psychiatric disorders [34]. Furthermore, while cutting-edge machine learning algorithms can be applied with the final aim of identifying potential biomarkers for psychiatric disorders [35–39], these new methodologies are not free from limitations [40]; thus, the results obtained are not reliable enough to be applied in the clinical context [41].

The nature of developmental and acquired pedophilia is thus widely different, as developmental pedophilia is a psychiatric disorder, while acquired pedophilia is a symptom of an underlying neurological disorder. Therefore, their etiology differs significantly. The etiology of developmental pedophilia is still unknown, but it is thought to be multifactorial [42–44], with genetics, stressful life events, testosterone exposure, neurochemical impairment and comorbidity with psychopathology all playing a pivotal role; this is in contrast to the etiology of acquired pedophilia, which is known and depends on the underlying neurological disorder. For instance, if the underlying neurological disorder is a brain tumor, then the etiology is neoplastic, while if the underlying condition is frontotemporal dementia, then the etiology is degenerative. This differentiation clearly impacts treatments; while developmental pedophilia is the primary disorder, both pharmacological and nonpharmacological treatments should be effective on pedophilia itself [45, 46]. Different is the case of acquired pedophilia, which is a symptom of a neurological disorder. In this case, treatments should be effective on the underlying neurological disorders, as it has been shown that, when possible, pedophilic tendencies and urges recede if the neurological disorder is treated. In these cases, *a restitutio ad integrum* is possible [15, 16].

Another key difference between the two forms of pedophilic behavior relies on their neural basis. In addition to the macroscopic differences, neural alterations in acquired pedophilia are usually evident, while those of developmental pedophilia are subtle; however, nothing is known regarding neural networks. In other words, the brain regions in which the synergic functioning is abnormal in the two forms of pedophilia are unknown. Regarding idiopathic pedophilia, some studies have attempted to shed light on structural or functional alterations [42, 47–55], but a recent meta-analysis concluded that the results do not spatially converge across different studies [10]; in other words, different studies have reported different results. Thus, to date, there are no consistent subtle brain alterations associated with developmental pedophilia. Lesions causally linked to acquired pedophilia are heterogeneous within the brain as well. Indeed, acquired pedophilia can emerge following lesions in the frontal lobe [13, 15, 56, 57], globus pallidum [58] (cases 4 and 6), hippocampus [14, 58, 59], etc. However, despite being spatially heterogeneous within the brain, all these brain regions localize to a shared network as they are functionally linked to the same brain regions, namely, the orbitofrontal cortex bilaterally and the posterior midline structures, including the precuneus and the posterior cingulate cortex [10].

Despite recent advances in the understanding of this disorder [9, 10, 60], the behavioral and clinical characterization of acquired pedophiles, the contribution of neuroscientific methodologies to the diagnosis and differential diagnosis of developmental pedophilia and the consequences of misdiagnosis remain underinvestigated. Similarly, the forensic and legal implications remain controversial.

Although this seems a topic of secondary importance, given the rarity of acquired pedophilia, which is described in a limited number of cases within the scientific literature [10, 60], recent evidence suggests that acquired pedophilia prevalence might be higher than previously expected but may pass unrecognized as an in-depth neuroscientific investigation aiming to understand the origin of offenses toward children is very seldom performed [60]. It is thus of the utmost importance to clarify the behavioral and clinical characterization of acquired pedophiles to help clinicians clarify which behavioral red flags could raise the suspicion of an acquired origin of pedophilia. Furthermore, it would also be helpful to shed light on both the neural network potentially involved in the two different forms of pedophilia—which is a topic that has thus far remained underexplored [10, 42, 48]—and the neuroscientific methodologies that could be considered important for the differential diagnosis between acquired and developmental pedophilia. Finally, the levels of awareness between the scientific and nonscientific communities should be raised with regard to the consequences of misdiagnosis, and critically, a consensus within the scientific community should be achieved with regard to the legal implications of such a disease.

For these reasons, a consensus conference of experts on the topic using the Delphi method [61–63] was conducted, aiming to provide the scientific community and clinicians with recommendations on how to deal with individuals with pedophilia who committed sexual offenses toward children. Since the literature on acquired pedophilia is thus far scant, we needed a method able to go beyond the state of the art, which would help clarify not what is actually done but what could/should be done [61]. The final aim of this work was to improve the differential diagnosis between developmental and acquired pedophilia, to increase the possibility of avoiding the consequences of misdiagnosis and to give offenders the right form of medical and/or psychological treatment and the right form of punishment.

## Methods

### Preregistration

The protocol was registered in the International Prospective Register of Systematic Reviews (PROSPERO - Registration Number: CRD42020159459). This study follows the Guidance on Conducting and Reporting Delphi Studies (CREDES) [64] available within the Enhancing the Quality and Transparency of health research (EQUATOR) Network.

### Systematic literature search

A systematic search to update the previous search already performed by the authors [60] was conducted in accordance with the PRISMA guidelines [65]. Papers reporting cases, reviews and case collection and critical analysis were systematically searched within the literature. The search identified 21 papers reporting original cases of acquired pedophilia, for a total of 25 offenders [8, 11–18, 56–59, 66–73], 6 reviews and case collections [10, 42, 48, 60, 74–76] and 2 critical analyses of the ethical aspect of the disorder [9, 77]. The screening procedure is summarized in the PRIMSA flow chart presented in Supplementary Material A. All these papers were shared with the Delphi panelists prior to the beginning of the Delphi process to ensure that all the panelists shared the same background information. It is also important to emphasize that at the time of both the search and the consensus conference, one paper reporting aggregated data on already published cases of acquired pedophilia [10] and one single case [78] were not available yet.

### The Delphi method

The Delphi method consists of “a series of structured group processes, each referred to as a round, to survey expert opinion and reach a group response” [79]; thus, the objective of this method is the reliable and creative exploration and production of suitable information for decision-making through an exercise in group communication among a panel of experts [80]. This method enables effective decisions to be made in situations where there is contradictory or insufficient information [62].

In this paper, the Delphi method was used to create consensual guidelines on how to deal with suspected acquired pedophiles. The Delphi consensus process was conducted between 2019 and 2020. The whole procedure will be described in the following paragraphs. We defined a priori that, to reach agreement, each statement needed to be approved by ≥75% of the raters [62, 63, 81–83].

### Selection of Delphi panelists

Choosing the most appropriate Delphi panelists is a very important step because it is directly related to the quality of the results generated. Unfortunately, throughout the Delphi literature, the definition of Delphi panelists has remained ambiguous [61]. Regarding the criteria used to guide the selection of Delphi panelists, individuals are generally considered eligible to be invited to participate in a Delphi panel if they have somewhat related backgrounds concerning the target issue and are thus capable of contributing helpful inputs and willing to revise their initial or previous judgments for the purpose of reaching or attaining consensus [61, 63]. To this aim, the proponent group (authors from the University of Padova) contacted a restricted group of experts in the field of acquired pedophilia, defined as the authors of previously published cases. The corresponding author of each paper was thus contacted (Supplementary Material B). Thirteen experts agreed to participate in the Delphi panel. After obtaining the confirmation for commitment and before starting the statement definition process, authors from the University of Padova shared “must-know” literature on acquired pedophilia with the panelists.

Furthermore, 560 editors of relevant scientific journals related to the topic of acquired pedophilia (i.e., sexual medicine, neuropsychology, neurology and psychiatry) were also contacted and invited to participate in this consensus conference.

### Statements definition

The Delphi process traditionally begins with an open-ended questionnaire [61], which is useful to solicit specific information about the topic. After receiving the subject’s responses, the technique requires investigators to convert the information into a questionnaire consisting of well-structured questions/statements. To this aim, the authors (*n* = 13) of previously published case reports were asked to provide, through answering open-ended questions, their opinions on the following topics: behavioral indicators, neuroscientific investigations, neurological conditions potentially leading to acquired pedophilia, consequences of misdiagnosis, legal consequences, prejudice, and future directions. The form including the open-ended questions is presented within Supplementary Material C. As requested by the Delphi method, answers to these questions were shared among the experts/authors; these answers are presented within Supplementary Material D. Responses were subject to content analysis; based on the authors’ opinions, 72 statements were created by the authors from the University of Padova.

### Delphi recursive voting

In Round 1, experts were invited to evaluate the 72 statements created on the basis of their answers to the preliminary open-ended questionnaire. Particularly, experts were asked to rate their agreement with each statement by means of a Likert scale ranging from 1 (*strongly disagree*) to 5 (*strongly agree*); 3 was chosen as a “*neutral/I do not know*” response. Statements were divided into sections as follows: behavioral indicators (20 items); neuroscientific investigation (18 items); neurological condition (11 items); consequences of misdiagnosis (8 items); legal consequences (8 items); and issues and future perspectives (7 items). A statement was defined to be approved by each single rater if it was answered with a 4 or 5 Likert score (agree/strongly agree). Supplementary Material E includes both the initial email and the online form used in the first round of the Delphi panel. In this round, areas of disagreement and agreement were identified [61], using 75% agreement as the threshold. At the end of the survey, experts were given the possibility to make further comments or clarifications of their responses [61].

In Round 2, each panelist received a questionnaire including the items and ratings that did not meet the consensus during the first round. Items were included in round 2 only if the consensus was not reached because of the high percentage of “*I don’t know*” (=3) responses. In other words, if an item did not reach consensus because panelists rated the item as *“I strongly disagree*” (=1 or 2), the item was not reproposed during round 2. Each expert thus received a questionnaire including 13 “to be re-evaluated” items. In addition to providing agreement percentages to enable the authors’ awareness of the general opinion, these items were in some cases amended slightly for clarification according to feedback received, whereas in other cases, an explanation of the item was provided. Again, authors were asked to rate the items using the same 5-point Likert scale, as well as to explain why they disagreed or were unsure about a given item. The form of the second round is available within Supplementary Material F. A final questionnaire was thus created.

As a last step, this final questionnaire, as well as the relevant literature, was shared with the group of editors. The questionnaire began with four questions regarding the field of expertise, prior knowledge of the topic, their belief that pedophilia can emerge as a symptom of a neurological disorder, and their knowledge about the difference between acquired and developmental pedophilia. The editors were asked to rate their agreement with each item using the Likert scale. The percentage of agreement was obtained.

Each statement was included in the final recommendation only if the agreement was equal to or exceeded 75% for both experts and editors.

## Results

### Raters

A final number of 52 raters contributed to creating the final recommendations. In addition to the 13 experts (i.e., authors who have published cases of acquired pedophilia), 40 additional colleagues took part in the initiative. Of them, three are neurologists, five are psychiatrists, twelve are neuropsychologists, four are experts in ethics, seven are forensic experts, three are psychologists, one is a geriatrician, one is a neuroscientist, three are psychological sexologists, and one is a forensic psychologist.

Thirty-six of them (90%) declared that they were aware of the existence of acquired pedophilia even before receiving our invitation email and reading the literature suggested; 36 of them (90%) declared that they believe that pedophilia can emerge as a symptom of an underlying medical condition; and 39 of them (97.5%) reported knowing the difference between acquired and developmental/idiopathic pedophilia. The only colleague who claimed to not to be aware of such a difference was excluded from the analysis due to his or her failure to read the relevant literature we shared by email.

The results of the consensus conference are presented in Table 1. The final recommendations consist of 63 statements divided into six different topics.

**Table 1:** The table includes the final recommendations consisting of 63 statements divided into six different topics.

Statement		Expert Agreement first round	Second round reformulation	Expert Agreement Second round	Agreement within editors	Included in final recomendations
Behavioral indicators						
1	Acquired and developmental pedophilia are two widely different disorders/distinct entities	92.3%			82.6%	YES
2	Acquired pedophilia occurs “de novo”, i.e., in individuals who have never manifested pedophilic interests or urges before, determining a behavioral fracture.	92.3%			82.6%	YES
3	Unlike developmental pedophiles, acquired pedophiles behave in most cases with an impulse dis-control.	92.3%			78.3%	YES
4	The impulse dis-control typical of individuals suffering from acquired pedophilia might manifest as lack of premeditation, un-planned actions and absence of masking the sexual abuse.	84.6%			78.3%	YES
5	The impulse dis-control typical of individuals suffering from acquired pedophilia might have an impact on the physical places where abuses take place, as acquired pedophiles are likely to act in open or crowded spaces, where they can easily be seen.	61.6%	Not included within the second round		60.9%	NO
6	The presence of premeditation is an important behavioral indicator that discriminates between an acquired and a developmental pedophile, especially when premeditation manifests in grooming behaviors.	69.3%	The presence of premeditation is an important behavioral indicator that discriminates between an acquired and a developmental pedophile (acquired pedophiles do not premeditate)	77%	78.3%	YES
			Acquired pedophilia do not manifest grooming behaviors	84.6%	78.3%	YES
			While premeditation is always present in developmental pedophilia, acquired pedophiles might act without premeditating the sexual assaults	84.6%	78.3%	YES
7	Unlike individuals with developmental pedophilia, individuals with acquired pedophilia do not manifest a predatory behavior (i.e., active search for victims) but they are guided by occasional events.	77%			78.3%	YES
8	Unlike individuals with developmental pedophilia, individuals with acquired pedophilia show a lack of rumination (i.e., obsession, constant thought about their victims, etc.).	61.6%	Individual with developmental pedophilia usually show rumination (i.e., obsession, constant thought about their victims, etc.)	69.3%	n/a	NO
			Individuals with acquired pedophilia usually do not show rumination (i.e., obsession, constant thought about their victims, etc.)	77%	60.9%	NO
9	Acquired pedophilia can be characterized by the non-selective choice of the sexual partner, OR it can be associated with general hypersexuality.	84.6%			87%	YES
10	Unlike individuals with developmental pedophilia, individuals with acquired pedophilia do not usually show aggressive behavior during their abuses (physical violence other than the sexual one, coercion).	69.3%	Not included in the second round		47.8%	NO
11	Unlike developmental pedophiles, acquired pedophiles behave **in most cases** with an insufficient moral judgment, which prevents them from understanding the wrongness of their action	53.9%	Individuals with developmental pedophilia have spared moral judgement: i.e., they are aware of the wrongness of their action	61.6%	n/a	NO
			Individuals with acquired pedophilia **might have** impaired moral judgement	84.6%	87%	YES
			In the cases where moral judgement is impaired, individuals might not be aware of the wrongness of their actions	84.6%	87%	YES
12	The insufficient moral judgment **typical** of individuals suffering from acquired pedophilia might manifest as spontaneous confession or lack of sense of guilt, as they do not understand the moral and legal implications of their actions.	69.3%	The insufficient moral judgement that **might be present in** individuals suffering from acquired pedophilia might manifest as spontaneous confession or lack of sense of guilt	84.6%	78.3%	YES
			In the cases where moral judgement is impaired, individuals with acquired pedophilia do not understand the legal and moral disvalue of their actions	84.6%	87%	YES
13	The impulse dis-control and the moral judgement deficits might be dissociated in acquired pedophilia: acquired pedophiles might act only as a consequence of an irresistible impulse although they are aware of the moral, social and legal disvalue of their acts OR they are only unable to understand that their behavior is morally, socially and legally wrong.	92.3%			82.7%	YES
14	As acquired pedophilia occurs as a symptom of a neurological insult, it does not share with developmental pedophilia the psychological risk factors, as for instance having been abused during infancy or high comorbidity with other psychiatric disorders.	77%			78.3%	YES
15	While developmental pedophilia is usually present since adolescence, the age of the onset of acquired pedophilia is usually delayed due to its acquired origin.	100%			91.4%	YES
16	To evaluate the presence of acquired pedophilia, additional behavioral indicators or abnormalities, indicative of impulse dis-control or of impaired moral judgment, might be present in individual’s daily life habits and outside the modus operandi.	92.3%			87%	YES
17	A detailed anamnesis should be carried out in order to understand whether or not additional behavioral indicators (not legally relevant and present in the individual’s daily life) are present or not.	92.3%			100%	YES
18	Unlike developmental pedophiles, acquired pedophiles are likely to present concomitant focal cognitive alterations, as a consequence of the brain disorder that also causes acquired pedophilia.	92.3%			91.3%	YES
19	Unlike developmental pedophiles, acquired pedophiles are likely to present concomitant neurological symptoms and signs indicative of brain sufferance.	100%			87%	YES
20	Acquired pedophilia is a multifactorial phenomenon and for this reason a psychological tool should be developed to capture the subjective experience of acquired pedophilia.	84.6%			87%	YES
Neuroscientific investigation						
21	If acquired pedophilia is suspected, an in-depth neuro-scientific investigation is warranted to further explore this condition	100%			95.7%	YES
22	Acquired pedophilia cannot be the sole symptoms of an underlying brain insult, but additional behavioral alteration, neurological symptoms, etc., should be present as well.	92.3%			91.4%	YES
23	Most of the neuro-scientific evidence (i.e., brain scans, neurologic symptoms) cannot be malingered.	92.3%			78.3%	YES
24	Including a NEUROPSYCHOLOGICAL EXAMINATION within the neuro-scientific investigation, it can be helpful to discriminate between developmental and acquired pedophilia as individuals with acquired pedophilia might present neuropsychological deficits consistent with the underlying neurologic disorder.	100%			100%	YES
25	Neuropsychological tests measuring the ability to control impulses (such as the go/no go task, etc.) can be useful to investigate whether the impulse control component is spared or impaired. If the impulse component is impaired even in a neutral task, acquired origin of pedophilia might be suspected.	92.3%			82.7%	YES
26	Neuropsychological tests measuring moral judgement (such as the ability to discriminate right from wrong, to identify a normal behavior, to evaluate the severity of a behavioral violation, etc.) can be useful to investigate whether the moral component is spared or impaired. If the moral component is impaired, even in a neutral task, acquired origin of pedophilia might be suspected.	69.3%	Neuropsychological tests measuring moral judgement (such as the ability to discriminate right from wrong, to identify a normal behavior, to evaluate the severity of a behavioral violation, etc.) can be useful to investigate whether the moral component is spared or impaired	92.3%	78.3%	YES
			If the moral component is impaired, even in tasks that do not investigate sexual behaviors, acquired origin of pedophilia might be suspected	92.3%	78.3%	YES
27	Neuropsychological impairment can potentially be malingered. Thus, particular attention should be paid to this. Neuropsychological tests accounting for malingering of impulse discontrol and impairment in moral judgement should be developed and used.	92.3%			82.6%	YES
28	Including PSYCHIATRIC ASSESSMENT within the neuro-scientific investigation it can be helpful to discriminate between developmental and acquired pedophilia as acquired pedophiles are not expected to show high comorbidities with other psychiatric disorders (in particular with personality disorders), differently from developmental pedophiles.	69.3%	Including PSYCHIATRIC ASSESSMENT within the neuro-scientific investigation it can be helpful to discriminate between developmental and acquired pedophilia	84.6%	78.3%	YES
29	Including a NEUROLOGICAL ASSESSMENT within the neuro-scientific investigation it can be helpful to discriminate between developmental and acquired pedophilia, as acquired pedophilia might be associated with signs and symptoms typical of the underlying neurological disorder.	100%			95.7%	YES
30	Neurologic signs of frontal lobe dysfunction can be a key characteristic of acquired pedophilia.	92.3%			87%	YES
31	Including NEUROIMAGING within the neuro-scientific investigation it can be helpful to discriminate between developmental and acquired pedophilia as acquired pedophilia should be originated by a neurological disorder usually visible at brain scan (unless in its very early stage).	100%			91.4%	YES
32	Brain imaging should be coupled with behavioral diagnosis in order to make the diagnosis of acquired pedophilia as reliable as possible.	100%			100%	YES
33	Brain insult leading to acquired pedophilia should be clearly evident. In other words, subtle brain abnormalities emerging only after a statistical analysis of the brain scans (for instance using Voxel Based Morphometry, etc.) could not be used as evidence supporting the presence of acquired pedophilia. Indeed, psychiatric disorders (i.e., developmental pedophilia) might be characterized by subtle abnormalities as well.	61.6%	After explanation	84.6%	87%	YES
34	The brain pathology associated with the behavioral variant of fronto-temporal dementia (bvFTD) might not be clearly evident in the early stage of the disorder, making bvFTD an exception to the previous statement. However, the fast progression of the disorder and the possibility to support its diagnosis using alternative brain imaging methods (i.e., PET) can help in the diagnostic differentiation.	92.3%			78.3%	YES
35	Including PSYCHOPHYSIOLOGICAL INVESTIGATIONS, like heart rate, startle reflex and skin conductance, within the neuro-scientific examination can be helpful to determine the presence of acquired pedophilia as these techniques can be helpful to exclude psychopathic traits	23.1%	Not included in the second round		30.4%	NO
36	Hormonal or biological analysis might support the possible presence of acquired pedophilia. Genetic investigations (PRGN & TAU) reveal neurobiological risk factors of acquired pedophilia.	61.6%	Not included in the second round		21.8%	NO
37	The IMPLICIT ASSOCIATION TEST (a behavioral test based on the compatibility effect and the analysis of reaction times) could help to support the late onset of pedophilic urges.	61.6%	After explanation	69.2%	56.5%	NO
38	Questionnaires investigating sexual behaviors should be provided to clinicians working with at risk populations (for example to all patients with specific neurologic disorders like dementias, Hungtington’s disorder, Parkinson’s disorder etc.) to further explore the possible insurgence of pedophilic tendencies in these patients.	84.6%			78.3%	YES
Neurological condition						
39	Acquired pedophilia emerge as a symptom of brain disorder. Despite acquired pedophilia has been described following brain tumor, traumatic injuries, surgical lesions, encephalitis, multiple sclerosis, dementias, etc, theoretically, pedophilia can occur as a symptom of any brain disorder.	61.6%	Acquired pedophilia emerge as a symptom of brain disorder. Despite acquired pedophilia has been described following brain tumor, traumatic injuries, surgical lesions, encephalitis, multiple sclerosis, dementias, etc., theoretically, acquired pedophilia can potentially occur as a symptom of a large variety of neurological disorder with different etiology	92.3%	91.4%	YES
40	Regardless the specific etiology of the underlying neurological insult, it is of the utmost importance to determine a strong temporal link between the onset of the neurological insult and the insurgence of the pedophilic tendencies.	84.6%			87%	YES
41	The brain network involved in pedophilia is still unknown and needs to be further investigated. Of note, the relevance of connections/ disconnections to the frontal lobe should be specifically assessed.	84.6%			95.7%	YES
42	Despite the brain network involved in acquired pedophilia is still unknown, any lesion affecting hypothalamus can potentially cause acquired pedophilia, as some nuclei of the hypothalamus are relevant for sexual orientation.	53.9%	Despite the brain network involved in acquired pedophilia is still unknown, lesions affecting the hypothalamus **can potentially contribute to the insurgence** (or: can potentially influence the insurgence) of acquired pedophilia, as some nuclei of the hypothalamus are relevant for sexual orientation.	92.3%	87%	YES
43	Despite the brain network involved in acquired pedophilia is still unknown, any lesion affecting the limbic system can potentially cause acquired pedophilia, as the limbic system is relevant for sexual behaviors and emotions.	69.3%	Despite the brain network involved in acquired pedophilia is still unknown, lesions affecting the limbic system **can potentially contribute to the insurgence (or: can potentially influence the insurgence)** of acquired pedophilia, as the limbic system is relevant for sexual behaviors and emotions	100%	87%	YES
44	Despite the brain network involved in pedophilia is still unknown, any lesion affecting orbitofrontal cortex can potentially cause acquired pedophilia, as the orbitofrontal cortex is relevant for impulse control.	84.6%			82.6%	YES
45	Despite the brain network involved in pedophilia is still unknown, any lesion affecting ventro-medial and/or dorso lateral prefrontal cortex can potentially cause acquired pedophilia, as these brain regions are relevant for moral judgement.	77%			78.3%	YES
46	Besides disorders with specific etiology, acquired pedophilia can also emerge due to biochemical imbalance (e.g., addiction to dopaminergic drugs to treat Parkinson’s disease).	77%			78.3%	YES
47	Deep brain stimulation for movement disorders may result in hyper-sexuality, impulse control disorders and disinhibition, which may increase the risk of pedophilic behavior.	61.6%	Deep brain stimulation for movement disorders may result in hyper-sexuality, impulse control disorders OR disinhibition, which may increase the risk of pedophilic behavior	69.2%	43.5%	NO
48	Epidemiological studies are needed to be able to better evaluate the prevalence and incidence of acquired pedophilia.	92.3%			91.3%	YES
49	To date, pedophilia and other paraphilias are the only psychiatric disorders within the DSM 5 that do not include the diagnostic criteria: «the symptoms are not attributable to the physiological effects of a substance or another medical conditions». As acquired pedophilia is a clear entity, we suggest this criterion should be added to pedophilia within the next DSM edition.	92.3%			82.6%	YES
Consequences of misdiagnosis						
50	It is important to differentiate developmental from acquired pedophilia as the two require different treatments and/or rehabilitation trajectories.	100%			95.7%	YES
51	It is important to correctly differentiate between individuals with acquired versus developmental pedophilia, because acquired pedophiles often need medical treatment to both treat the underlying neurological disease and to arrest the pedophilic tendency.	100%			87%	YES
52	The misdiagnosis of acquired pedophilia might have dramatic consequences for the sexual offender as acquired pedophilia often has a treatable etiology. Thus, a misdiagnosis has an impact on the defendant’s health (and possibly on his life).	100%			95.7%	YES
53	As acquired pedophilia often origins from a treatable underlying disorder and a “restitutio ad integrum” has been described following treatment of the underlying neurological condition, a correct diagnosis might help in preventing further sexual offenses/recidivism.	100%			82.6%	YES
54	The consequences of the misdiagnosis of acquired pedophilia are potentially severe for the defendant’s family, that is suffocated by social stigma. The right diagnosis might help relatives to have a rational explanation of their relative’s behavior. Indeed, from the psychological point of view it is very different to consider a relative as a sexual predator or, on the contrary, as a patient with a neurological disorder producing involuntary inappropriate behavior.	100%			95.7%	YES
55	The misdiagnosis of acquired pedophilia might potentially have ethical consequences, as it is an ethical concern to put in jail someone who has a life-threatening condition impacting on his behavior and who would benefit more from medical treatment. In these cases, the offenders should be in any case closely monitored to avoid re-offending.	100%			95.7%	YES
56	The misdiagnosis of acquired pedophilia might potentially have ethical consequences, as it would be unethical to impose an inappropriate legal solution based on a retributive penalty instead of a rehabilitative option.	92.3%			100%	YES
57	Attention should be paid to the risk of stigmatization of both developmental and acquired pedophilia. As there are pedophiles who never committed sexual crimes and who are actively asking for help, their stigmatization should be avoided as it will not help in preventing crimes.	92.3%			87%	YES
Legal consequences						
58	A case-by-case approach is the most appropriate when establishing the legal consequences of acquired pedophilia.	84.6%			91.4%	YES
59	The legal consequences on insanity should be different between developmental and acquired pedophilia.	84.6%			78.3%	YES
60	While defendants with developmental pedophilia are to be considered criminally liable, defendants presenting with acquired pedophilia can be considered not guilty by reason of insanity.	61.6%	Defendants with developmental pedophilia are to be considered criminally liable	92.3%	95.7%	YES
			Defendants presenting with acquired pedophilia can be considered not guilty by reason of insanity	77%	87%	YES
			It is relevant to assess insanity in individual with acquired pedophilia	92.3%	87%	YES
61	When acquired pedophilia is suspected, the presence of an underlying neurological condition is not per se enough to lead to insanity, but the impact of the neurological condition on relevant behaviors (i.e., moral reasoning and impulse control) should be carefully assessed.	84.6%			100%	YES
62	Acquired pedophiles should be assigned to a non-reclusive condition, however they should be treated in places where their social danger is neutralized until recovery.	100%			91.4%	YES
63	Acquired pedophiles should not be condemned to jail, as jail restriction is inadequate as they need to be treated rather than/before being punished.	84.6%			95.7%	YES
64	Alternative structures to jail (hospices or something else) may be evaluated for acquired pedophiles.	92.3%			87%	YES
65	Determining whether the individual suffering from acquired pedophilia had pre-morbid sexual interests towards children but never acted them, is a scientifically interesting question. However, it is not legally important, as individuals should be judged from their behaviors.	84.6%			78.3%	YES
Issues and future perspectives						
66	There is definitively a prejudice within the scientific community on this topic.	84.6%			78.3%	YES
67	The existing prejudice on this topic will be very hard to overcome. Indeed, providing an explanation is considered equivalent to providing a justification.	77%			78.3%	YES
68	The only way to diminish the prejudice on this topic is to publish and disseminate the results of research, to provide a better explanation of how the brain disorders can lead to acquired pedophilia, which are the associated behavioral indicators, and how can sexual offense be prevented in these patients.	84.6%			82.6%	YES
69	The only way to overcome prejudice is to keep people informed with the latest scientific results: acquired pedophiles, despite they have committed a crime, are patients that need medical treatment.	100%			82.6%	YES
70	It will be useful to include the perspective of victims in any paper concerning criminal justice responses to acquired and developmental pedophilia. Too often, the perspective of victims is not addressed. Victims often have a need to understand why an individual has committed a particular crime.	84.6%			69.6%	NO
71	Neuropsychology teaches us that every cognitive component might be selectively damaged. Sexual behavior is a complex function that requires the integrity of many cognitive components (gender recognition, age estimation, moral reasoning, theory of mind, impulse control, to name a few) in order to be carried out. It is thus unreasonable to believe that sexual behavior, being the result of many complex cognitive tasks, could not be affected by neurological damage.	100%			91.4%	YES
72	Further studies should try to better estimate the prevalence and incidence of acquired pedophilia, by systematically surveying a consecutive series of individuals charged or convicted with pedophilic activities in a defined region (e.g., country or state).	100%			91.4%	YES

*n/a* not applicable.

### Behavioral indicators

The panel agreed on 17 statements on behavioral indicators or red flags that can be helpful for determining the suspicion level of being in the presence of a case of acquired pedophilia. The final recommendations suggest that developmental and acquired pedophilia are two different disorders, with acquired pedophilia occurring “*de novo*” in individuals who have never manifested pedophilic interests or urges before, thereby determining a behavioral fracture.

The *modus operandi* of individuals with acquired pedophilia is considered to be impulsive; this differentiates them from individuals with developmental pedophilia. This lack of impulse control might manifest as lack of premeditation, unplanned actions, and the absence of masking sexual abuse. In particular, the presence of premeditation is an important indicator that discriminates between acquired and developmental pedophilia, as acquired pedophiles usually do not premeditate their offenses and do not manifest grooming behaviors. Consequently, they are guided by occasional events and do not actively search for victims; this is in contrast to developmental pedophiles, whose *modus operandi* is predatory. For the same reason, acquired pedophiles do not selectively choose their sexual partners and can manifest general hypersexuality.

In addition to a lack of impulse control, individuals with acquired pedophilia might have an impaired moral judgment that might manifest as spontaneous confession or lack of sense of guilt; in these cases, they might not be aware of the wrongfulness or the legal and moral disvalue of their actions.

Importantly, the lack of impulse control and deficits in moral judgment might be dissociated in these offenders. The presence of both of these behavioral abnormalities should be evaluated not only in the analysis of the *modus operandi* but also in the daily life of the offenders. For this reason, a detailed anamnesis is invaluable.

Regarding the criminal profiling, as acquired pedophilia occurs as a symptom of a neurological insult, this form of pedophilia does not share with developmental pedophilia the same risk factors, i.e., having been abused during infancy or having high comorbidity with other psychiatric disorders. For the same reason, this condition is not present in individuals since adolescence; rather, its onset is usually delayed due to its acquired origin. However, acquired pedophiles are likely to present concomitant focal cognitive alterations (i.e., deficits in memory) and neurological symptoms and signs (i.e., abnormal reflexes) as a consequence of the underlying brain disorder.

Finally, we suggest that acquired pedophilia is a multifactorial phenomenon; for this reason, we further suggest that a psychological tool should be developed to capture the subjective experience of acquired pedophilia.

### Neuroscientific investigation

The panel agreed on 14 statements regarding the neuroscientific investigation that is recommended to be performed in cases of suspected acquired pedophilia. Neuroscientific examinations are important, as in most cases, their results cannot be malingered.

First, it is important to carry out an in-depth neuropsychological examination. This is because acquired pedophilia cannot be the only symptom of the underlying brain insult; thus, a cognitive evaluation could be helpful in identifying the presence of additional functional/cognitive deficits consistent with the underlying neurologic disorder. Such neuropsychological evaluation is recommended to focus on the ability to control impulses (i.e., go/no go task) and on the ability to engage in social cognition (i.e., ability to discriminate right from wrong, ability to identify behavioral violations and to graduate their severity). In both cases, if the cognitive functions are impaired even in neutral tasks (i.e., task not involving the sexual component toward children), then acquired pedophilia might be suspected. Since neuropsychological results could potentially be malingered, the scientific community is recommended to develop and validate tests to account for the presence of malingering for impulse control and social cognition.

In addition to the neuropsychological evaluation, psychiatric and neurologic evaluations are also recommended, as they can be helpful to differentiate between developmental and acquired pedophilia. The latter is important for the identification of signs and symptoms typical of the underlying neurological disorder. Particular relevance should be paid to neurologic signs of frontal lobe dysfunction.

Finally, the use of neuroimaging techniques is also strongly recommended. Experts, however, recommend caution, as brain imaging findings should always be coupled with behavioral symptoms and diagnosis to make the diagnosis of acquired pedophilia as reliable as possible. In other words, the panel all agreed that the presence of a neurological condition and pedophilic behavior is not sufficient to make the diagnosis of acquired pedophilia if additional signs and symptoms are absent. Two additional cautionary statements were also approved. The first one recommends that the brain insult leading to acquired pedophilia should be clearly evident. This is important, as in the last few decades, methods and algorithms used to identify subtle brain abnormalities have been developed and become widely used. However, these subtle brain abnormalities should not be used as evidence supporting the presence of acquired pedophilia, as subtle abnormalities could also be present in developmental pedophilia. The second cautionary statement recommends that particular attention should be given to the case of acquired pedophilia emerging as a symptom of behavioral variants of frontotemporal dementia. In this case, clearly evident brain abnormalities could be absent, particularly in the early stage of the disorder. Thus, bvFTD is an exception to the previous statement, unless alternative brain imaging techniques (i.e., positron emission tomography) are used to confirm the underlying neurological diagnosis. These patients should be followed-up, as bvFTD is characterized by a fast progression.

Finally, we also recommend that questionnaires investigating sexual behaviors should be provided to clinicians working with at-risk populations to further explore the possible insurgence of pedophilic tendencies in these patients. This recommendation is particularly useful to try to prevent sexual offenses.

### Neurological conditions

The panel agreed on 10 statements regarding the neurological conditions that can be linked to the insurgence of acquired pedophilia. Acquired pedophilia can indeed emerge as a symptom of a large variety of neurological disorders. Despite the specific etiology, it is of the utmost importance to determine a strong temporal link between the onset of the neurological disorder and the insurgence of the pedophilic urges. In addition, epidemiological studies are needed to better evaluate the prevalence and incidence of acquired pedophilia.

The brain network impairment in acquired pedophilia is still unknown and needs to be further investigated. We recommend that the disconnection to/from the frontal lobe should be specifically assessed in future research. Indeed, lesions affecting the orbitofrontal cortex and the ventro-medial and/or dorsolateral cortex can potentially influence the insurgence of acquired pedophilia as well, as the former is relevant for impulse control, while the latter is relevant for moral judgment. Similarly, lesions affecting the limbic system and the hypothalamus can potentially influence the insurgence of acquired pedophilia. Indeed, normal functioning of the limbic system is relevant for sexual behavior and emotions, and some nuclei of the hypothalamus are known to be relevant for sexual orientation.

In addition to disorders with a specific etiology, acquired pedophilia can also emerge due to biochemical imbalance. An example available within the literature is the addiction to dopaminergic drugs to treat Parkinson’s disease.

It is of critical importance to note that the panel agreed that, to date, pedophilia and other paraphilias are the only psychiatric disorders within the DSM 5 that do not include the following diagnostic criteria: “*the symptoms are not attributable to the physiological effects of a substance or another medical conditions*”. As acquired pedophilia is a clear entity, the panel consensually suggested that this criterion should be added to the pedophilia section within the next DSM edition.

### Consequences of misdiagnosis

The panel agreed on 8 statements regarding the consequences of misdiagnosis. Indeed, the differentiation between developmental and acquired pedophilia is important, as they require different treatments and/or rehabilitation trajectories, as the latter often has a treatable etiology. Thus, acquired pedophiles often need medical treatment of the underlying neurological condition; this treatment can potentially lead to a remission of the pedophilic tendencies, thus helping to prevent further sexual offenses/recidivism. In addition, receiving appropriate medical treatment can have a positive impact on a patient’s health and life.

Critically, the consequences of misdiagnosis are not only medical but also ethical. Indeed, these offenders’ families are often suffocated by social stigma. For this reason, giving the right interpretation of the offense might help families to have a rational explanation of their relative’s behavior. From the psychological point of view, it is very different to consider a relative as a sexual predator or, in contrast, as a patient with a neurological disorder that produces involuntary inappropriate behavior. In addition, another ethical concern due to potential misdiagnosis is related to the consequences for the defendant; it is an ethical concern to put in jail someone who has a life-threatening condition that impacts his or her behavior and who would benefit more from medical treatment. Thus, it would be unethical to impose an inappropriate legal solution based on a retributive penalty instead of a rehabilitative option. In these cases, the offenders should in any case be closely monitored to avoid reoffending.

A final important ethical concern that needs attention is related to the risk of stigmatization of both developmental and acquired pedophilia (not pedophilic behavior). As there are pedophiles who have never committed sexual crimes and who are actively asking for help, their stigmatization should be avoided, as such stigmatization will not help in preventing crimes.

### Legal consequences

The panel agreed on 8 statements regarding the potential legal consequences for individuals with acquired pedophilia. Although we agreed that a case-by-case approach is the most appropriate when establishing the legal consequences of acquired pedophilia, we also believe that different legal consequences are warranted for individuals with developmental and acquired pedophilia; while individuals with developmental pedophilia are almost always to be considered criminally liable, insanity should be carefully assessed in individuals with acquired pedophilia, as they can be considered not guilty by reason of insanity. During insanity evaluation, the presence of an underlying neurological condition should not be considered per se enough for insanity; rather, the impact of the neurological condition on the individual’s relevant behavior (i.e., ability to withhold actions and ability to understand the legal and moral disvalue of offenses) should be carefully assessed.

Acquired pedophiles should not be condemned to jail; jail restriction is inadequate, as these individuals need to be treated rather than/before being punished. Instead, they should be assigned to nonreclusive structures, where they can be treated and where their social danger can be neutralized until recovery.

Critically, some acquired pedophiles admit they had sexual interest toward children even before the insurgence of their neurological condition, but they never acted on these tendencies. This should not be considered legally relevant, as individuals should be judged based on their behaviors and their choices. If they felt the urges previously but chose not to act on them, and after the insurgence of the neurological condition, they are no longer able to make this choice, this means that the neurological insult led to a behavioral fracture due to disinhibition and/or a deficit in the understanding of the moral and legal wrongfulness of the actions.

### Issues and future perspectives

The panel agreed on 8 statements regarding issues and future perspectives. Importantly, the panel recognized that there is definitively a prejudice, which will be very hard to overcome, within the scientific community on this topic. We consensually believe that the only way to diminish this prejudice is to increase the number of publications and disseminate the results of the related research to provide a better explanation of how brain disorders can lead to acquired pedophilia, which are the associated behavioral indicators, and how sexual offenses can be prevented with regard to these patients. In other words, our mission should be to keep people informed of the latest scientific results; i.e., acquired pedophiles, despite having committed a crime, are patients who need medical treatment.

Indeed, neuropsychology teaches us that every cognitive component might be selectively damaged [84]. Sexual behavior is a complex function that requires that the integrity of many cognitive components (gender recognition, age estimation, moral reasoning, theory of mind, impulse control, to name a few) be carried out. It is thus unreasonable to believe that sexual behavior, which is the result of many complex cognitive tasks, could not be affected by neurological damage.

Further studies should try to better estimate the prevalence and incidence of acquired pedophilia by systematically surveying a consecutive series of individuals charged or convicted with pedophilic activities in a defined region (e.g., county or state).

## Discussion

To date, the scientific and clinical communities are still devoid of clear guidelines on how to evaluate patients with pedophilia/pedophilic behavior. Indeed, it is still very difficult to discriminate whether pedophilic tendencies are of psychiatric origin or rather reflect the insurgence of an underlying neurological disorder. Although a recent paper suggests the possibilty of a behavioral profiling of patients with acquired pedophilia [60], consensus is still lacking between experts.

The current consensus conference aimed to assist clinicians in the difficult differential diagnosis between developmental and acquired pedophilia by providing step-by-step guidelines that could be followed to decrease the risk of misdiagnosis. The Delphi process addressed six areas of uncertainty concerning the diagnostic iter for acquired pedophilia. Overall, consensus was reached for 63 out of 72 initial statements. Unfortunately, the data available within the literature are very scant due to the supposed rarity of the condition, and there is a desperate need for evidence-based information. In addition to a few exceptions [10, 60], the available papers only describe single cases, and the description of behavioral red flags or biological markers is sometimes largely insufficient, with the result that conclusions on these cases are sometimes controversial and inadequate to properly identify a common nature of these patients.

The current consensus conference, capitalizing on the experience of the experts involved, was able to reach some important recommendations. In particular, behavioral red flags were identified, the most important of which was impulsivity (92.3% agreement). These red flags are particularly relevant, as they are reflected by the daily life behavior of the patients (92.3% agreement), in addition to their *modus operandi*. They are thus easily deducible from an accurate anamnesis and analysis of the offenders’ *modus operandi* (92.3% agreement).

Once the acquired origin of pedophilia is suspected, we have provided clinicians with recommendations on the following steps that are important for the correct classification of the disorder. In particular, we suggest performing an accurate neuropsychological evaluation (100% agreement) to have an accurate measure of the individual’s impulse dyscontrol and moral judgment impairment; performing a neurological evaluation (100% agreement) to identify the associated neurological symptoms; and performing a neuroimaging investigation to identify and clarify the brain insult (100% agreement). This is particularly relevant to understanding whether the underlying neurological condition leading to pedophilia is reversible. The consensus is slightly lower for the importance of performing a psychiatric evaluation (84.6% agreement); the scant literature available thus far seems to suggest that acquired pedophiles do not show comorbidities with other psychiatric disorders, particularly personality disorders (for a review see [60]). This could be a possible difference between acquired and developmental pedophilia, in which this comorbidity is widely acknowledged [45, 85, 86].

Regarding the neurological conditions that could lead to acquired pedophilia, pedophilia has been described as a symptom of brain tumors [15, 56], notochord tumors [16, 70], traumatic injury [13, 66], surgical lesions [58], multiple sclerosis [17], Hungtinton’s disease [58], hippocampal sclerosis [11], and behavioral variants related to temporal dementia [11, 12, 59]. However, the panel experts agreed that these conditions can potentially occur as symptoms of a large variety of neurological disorders (92.3% agreement). The neurological insult leading to pedophilia, thus, could have different etiologies: neoplastic, traumatic, surgical, degenerative, inflammatory, etc. Similarly, brain lesions leading to acquired pedophilia are heterogeneously widespread in the brain and include the orbitofrontal cortex [15, 16], ventromedial prefrontal cortex [13, 77], hypothalamus [8, 16], thalamus [8], hippocampi [11, 14], amygdalae [14], right globus pallidus [58], caudate [58], putamen [58], and striatum [58]. The mechanism explaining the insurgence of pedophilia from described brain lesions is not known thus far; however, a recent study suggests the emerging idea that all the lesions of the patients available thus far within the literature, despite being spatially heterogeneous in the brain, are localized to a shared network including the orbitofrontal cortex and the posterior midline structures (posterior cingulate cortex and precuneus) [10]. Using the functional characterization approach, these brain regions have been found to be particularly important for both impulse control and theory of mind [10]. In accordance with the INUS (insufficient but not redundant parts of unnecessary but sufficient conditions) model of causation [87], these results suggest the intriguing idea that both impulse control and theory of mind should be concomitantly impaired by the lesion to lead to acquired pedophilia. This idea should be tested in future studies. For this reason, a comprehensive assessment of these abilities is even more important. The current consensus conference did not recommend any specific neuropsychological test that could be used with this purpose; however, it is important to mention that impulse control abilities could be measured using tests such as the Stroop test [88], the Hayling test [89, 90], or the computerized go/no-go [91] or stop signal [92] tests. The affective variant of the stop signal task is also particularly interesting as it can reveal a difficulty in inhibition only when an emotional component in the stimuli is present [93]. Similarly, the theory of mind ability could be measured by means of the Story Empathy Task (SET, [94]), the Theory of Mind Inventory [95] or the social cognition battery [96].

To our knowledge, the consequences of misdiagnoses for individuals with acquired pedophilia have never been systematically investigated before [9]. The related sections thus represent a clear advance compared to the actual state of the art on this topic. Indeed, the recommendations suggest that a large prejudice on this topic is present within the scientific community (84.6% agreement); however, this line of research should continue and be improved as the consequences of misdiagnosis could be detrimental for both the patient and his family. It should be kept in mind that acquired pedophilia is a potential reversible condition; thus, the right diagnosis could lead not only to providing the patient with the right treatment but also to a remission of the whole symptomatology, including pedophilic tendencies (e.g., [15, 16]). In the case of acquired pedophilia, thus, the right diagnosis could be the most effective way to prevent recidivism (100% agreement). For this reason, the recommendations also highlighted that the legal consequences for these individuals should not be the same as those for individuals with developmental pedophilia (84.6% agreement).

Our recommendations stem from the scant available literature, as well as our shared multidisciplinary clinical experience, including neurology, psychiatry, psychology, neuropsychology, and ethics. Due to concerns regarding the potentially acquired nature of pedophilia in a few rare individuals, including the ethical and legal potential consequences, we emphasize the need for an “individualized” approach to the patient (84.6% of agreement) to strictly avoid the dangerous dichotomy of the “presence of an underlying neurological disorder = acquired pedophilia”. Indeed, it is widely known that evident brain lesions do not always lead to clinically relevant behavioral deficits. One example is the Italian case of the serial killer Gianfranco Stevanin who, despite the large lesion of traumatic origin within the frontal lobe, was perfectly able to withhold his impulses [97]. In contrast, we suggest that to support the acquired nature of pedophilia, we need to indisputably identify (i) the de novo insurgence of uncontrollable pedophilic urges determining a behavioral fracture (92.3% of agreement); (ii) impulsivity manifested in daily life (92.3% of agreement); (iii) impulsivity manifested in the *modus operandi* (92.3% of agreement); (iii) an underlying neurological disorder with associated neurological symptoms (100% of agreement); (iv) the insurgence of pedophilia temporally linked to the insurgence of the underlying neurological condition (84.6% of agreement); and (v) a neural basis in anatomo-clinical correspondence with the behavior of the patient (100% of agreement). If these conditions are present, there is a scientific basis to support the acquired nature of pedophilia; thus, insanity could become an important topic to explore (92.3% agreement).

This work is not free from drawbacks.

Despite our wide invitation to participate at this consensus conference, the percentage of invited scientists who agreed to participate was below 10% (40 out of 560 = 7.14%). This seems to be a common problem within this kind of study [61, 63, 98, 99]. In this case, the low participation rate could have two nonmutually exclusive explanations. On the one hand, we contacted the editors by email; thus, a hypothesis is that the emails were not received/read. On the other hand, we are aware that the topic of this consensus is extremely delicate; thus, some people might not be willing to give their personal opinion on a topic such as pedophilia. Of note, previous Delphi studies have been published considering a similar or lower number of panelists [100–102]. The literature also acknowledges that a low number of participants could be sufficient to reach reliable recommendations [63, 103]. In particular, one study of health care quality and safety used bootstrap sampling to investigate the stability of response characteristics and found that a panel of 23 experts produced stable results [100]. Given the nature of the topic, we believe we have been able to build solid knowledge in the current study.

Furthermore, in this consensus conference, we did not investigate the important topic of pharmacological and nonpharmacological treatments. The reason is that both pharmacological and nonpharmacological treatments strongly depend on the specific etiology of the underlying neurological disorder, for which clinicians already have clear, personalized guidelines. However, regarding the difference between developmental and acquired pedophilia, we acknowledge that the two forms of pedophilia have enormously different internal states, as well as risks and consequences for society; for this reason, they must be treated under different conditions. It is likely that acquired pedophilia could benefit from cognitive rehabilitation focused on insight, awareness, and recognition of legal and moral issues. On the other hand, developmental pedophilia, which is known to respond poorly to cognitive and psychotherapeutic treatments [46, 104] and have low levels of compliance [45, 86, 105], might benefit more from biochemical interventions [106, 107].

Similarly, in this consensus conference, we did not specifically expand the role of pharmacological treatment on iatrogenic pedophilia and its prevalence in neurological patients. To our knowledge, only two single cases of iatrogenic pedophilia have been published describing two patients with Parkinson’s disease. The first case manifested pedophilia as a consequence of the addition of pramipaxole [58]. The second case manifested hedonistic homeostatic dysregulation and pedophilia following the self-administration of an extra dose of dopaminergic drugs (especially pergolide) [71].

Third, in this paper, we considered acquired pedophilia to be the result exclusively of neurological brain disorders. However, recent evidence in animal models suggests that associative learning under the effect of enhanced dopaminergic activity might result in learned pedophilic-like sexual responses even in animals without brain damage [108]. These learned responses seem to depend on context, as they are observed only in animals in which dopamine administration was coupled with cohabitation with juvenile males. These results suggest that other factors, from potential genetic predisposition to epigenetic experience and enhanced D2 agonism, may play an etiological role in pedophilic interest insurgence and thus pedophilia could also emerge as a consequence of D2 enhanced agonism, for example.

A final remark is important. As already stated within the introduction, pedophilia and other paraphilias remain the only psychiatric disorders within the DSM-5 that do not include the following diagnostic criteria: *“the symptoms are not attributable to the physiological effects of a substance or another medical conditions”* [2]. Given the evidence now available [10, 60], including the current paper, we consensually suggest that this criterion should be added to the pedophilia section in the next DSM edition. Future research is needed to understand whether the concept of acquired pedophilia could be extended to “acquired paraphilias”.

To conclude, this article provides a comprehensive description of issues that may be of interest to psychiatrists, neurologists and forensic experts with regard to approaching such a delicate topic. Importantly, we strongly recommend promoting the translational implementation of the recommendations proposed in this consensus statements to other professionals working in different, complementary fields, such as judges and lawyers.

## Supplementary information

Supplementary Material A

Supplementary Material B

Supplementary Material C

Supplementary Material D

Supplementary Material E

Supplementary Material F
