# Supplementary material for: Acquired Pedophilia: international Delphi-method-based consensus guidelines
Source: Transl Psychiatry. 2023 Jan 18;13:11. doi: 10.1038/s41398-023-02314-8 (PMC9849353; doi:10.1038/s41398-023-02314-8)
Supplement: Supplementary file 1 — Supplementary Material A [file 41398_2023_2314_MOESM1_ESM.doc]

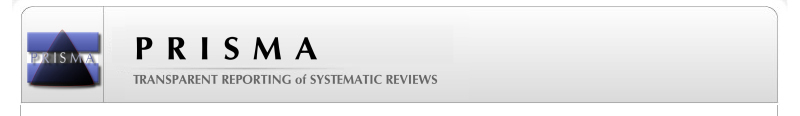
**PRISMA 2009 Flow Diagram**

**Acquired Pedophilia:**

**international Delphi-method-based consensus guidelines**

**Screening**

**Included**

**Eligibility**

**Identification**

Records identified through database searching
(n = 43)

Additional records identified through other sources
(n = 17)

Records after duplicates removed
(n = 58)

Records screened
(n = 58)

Records excluded
(n = 23)

Full-text articles assessed for eligibility
(n = 35)

Full-text articles excluded, with reasons

(n=5: not on acquired pedophilia;

n=1: not clear whether the pedophilia is acquired )

Studies included in qualitative synthesis
(n = 29)
