# Supplementary material for: Acquired Pedophilia: international Delphi-method-based consensus guidelines
Source: Transl Psychiatry. 2023 Jan 18;13:11. doi: 10.1038/s41398-023-02314-8 (PMC9849353; doi:10.1038/s41398-023-02314-8)
Supplement: Supplementary file 3 — Supplementary Material C [file 41398_2023_2314_MOESM3_ESM.docx]

**Pre-Delphi round: OPEN QUESTIONS:**

In the following pages, you will be asked to respond to 7 open questions, where you can express your opinion on a few aspects of acquired pedophilia.

We kindly ask you to answer carefully and thoughtfully, as these answers will be of critical importance for the construction of the next questionnaire.

Even if you do not feel your expertise is appropriate to answer these questions, feel free to contribute anyway.

If you feel like you can't or don't want to answer any of the following questions, type in "I don't know" or "I don't have anything to say" to proceed to the next question.

Once you have submitted the form, and you would like to add anything, feel free to start a new one. They will be both considered as one entry.

We would like to remind you that you have two weeks to answer.

Thank you for your collaboration.

**Question n.1 - Behavioral indicators**

A recent review of the literature conducted in accordance with the PRISMA guidelines identified differences in the modus operandi of acquired and developmental pedophilia. In particular, according with the analysis, acquired pedophiles are characterized by absence of premeditation, absence of masking their behavior, spontaneous confession and sense of guilt. Do you agree with these results? In your opinion, what is the most important behavioral difference between an acquired pedophile rather than a developmental one? Do you have some suggestion about additional behavioral indicators besides the one identified?

**Question n.2 - Neuroscientific investigation**

In our recent paper, we identified a possible behavioral profile of acquired pedophilia but we did not suggest any indication to support clinicians in the diagnostic process. In your opinion, how would it be possible to identify an acquired pedophile in neurological terms? Do you want to suggest any diagnostic tool or medical exam that might be helpful in the identification process? Do you think that it is possible to simulate this condition in any of these exams? What do you think is important to take into consideration for the best identification of an acquired pedophile?

**Question n.3 -Neurological condition possibly leading to pedophilia**

The systematic review of literature identified some neurological disorders that in rare cases might lead to pedophilia: i.e. brain tumor, frontotemporal dementia, etc. Are there any other cases you know of? Are you aware of any other medical condition that can cause pedophilic behavior? Do you think the actual research on the topic is adequate or do you think there are new important issues to be taken into consideration? In your opinion, how important can the correct identification of acquired pedophilia be for the research on neurological and psychiatric disorders?

**Question n.4 - Consequences of misdiagnosis**

The misdiagnosis of acquired pedophilia might have consequences both for the sexual offenders (some of the neurological disorders are life threatening conditions) and for the victims (being acquired pedophilia reversible in some cases, the correct identification might prevent further child abuses). Furthermore, there are ethical risks as well as it would be an ethical concern to put in jail someone who has a life threatening condition impacting on his behavior and who could benefit from a medical treatment. Do you agree/disagree with these opinions? If you disagree, can you explain why? In your opinion, what are the possible ethical and medical risks related to a missed identification of acquired pedophilia?

**Question n.5 - Legal Consequences**

There is an ongoing debate on the legal consequences of acquired pedophilia. Do you believe that the identification of acquired pedophilia should influence the legal process and/or have repercussions on the legal consequences in the forensic field, especially of imputability? In your opinion, should the criminal charges be different in cases of acquired and developmental pedophilia?

**Question n.6 – Prejudice**

When we submitted our manuscript for publication, we found extreme reticence in publishing the manuscript and concerns were expressed about publishing on the hypothesis of neurological insult causing pedophilic behavior. Do you believe there is any prejudice on this topic? If so, can you explain and give us some suggestions on how to diminish or remove this prejudice?

**Question n. 7 - Additional thoughts**

Is there anything else you would like to add on this topic that could be helpful for the present consensus conference? Please remember that this is a generative phase, anything you write might be important and helpful for this project.
