# Supplementary material for: Acquired Pedophilia: international Delphi-method-based consensus guidelines
Source: Transl Psychiatry. 2023 Jan 18;13:11. doi: 10.1038/s41398-023-02314-8 (PMC9849353; doi:10.1038/s41398-023-02314-8)
Supplement: Supplementary file 4 — Supplementary Material D [file 41398_2023_2314_MOESM4_ESM.docx]

**Responses to the open questions:**

**Email:**

Dear colleagues,

We would like to thank all of you for taking the time to participate in our project. In the attached file you will find the responses we have received from all of you.

We are now working on the statements based on these answers and the literature together.

If you would like any additional information about the project, please don't hesitate to contact us.

Thank you again.

Best regards

Cristina Scarpazza & Andrea Camperio Ciani

****************************************

**Question n.1 - Behavioral indicators**

A recent review of the literature conducted in accordance with the Prisma guidelines identified differences in the modus operandi of acquired and developmental pedophilia. In particular, according with the analysis, acquired pedophiles are characterized by absence of premeditation, absence of masking their behavior, spontaneous confession and sense of guilt. Do you agree with these results? In your opinion, what is the most important behavioral difference between an acquired pedophile rather than a developmental one? Do you have some suggestion about additional behavioral indicators besides the one we identified?

**Responses:**

- I agree with these results but believe it is not 'black and white', there are gradations in between. The acquired cases occur 'de novo' without hints of prior behaviors suggesting such pedofilic tendencies.
- For me it's the difference between instrumental vs. reactive behavior, lack of rumination, and often non-discerning sexual offending (meaning they often will be hypersexual with many types of victims, not just children).
- I do not agree. I am aware of at least one reported acquired pedophile who showed evidence of premedictation, masking behavior, and a sense of guilt. Rather I would proposition that the difference between acquired and developmental is the degree of impulse control that is involved, with the acquired being more of an impulse control issue, with the acquired having a greater impulse/opportunistic component. But that does not imply there is not awareness of the wrongness of the behavior. It is more of an inability to act on that awareness.
- The lack of a former interest in child in the acquired form. Absence of masking the behavior and spontaneous confessions can be associated also with psychiatric symptoms such as mania or a delusion. Sense of guilt can be considered a symptom of the acquired form only if more "moral" cerebral areas are not involved. Acquired paedophilia is a disorder that clearly change the former condition, in "standard" paedophilia the interest on child sexuality dates from adolescence or early youth.
- Yes.
- Yes, I Agree with these conclusions, in my opinion, the presence of premeditation is the most important behavioral indicator that discriminates between acquired and developmental pedophile. Especially when premeditation expresses in grooming behaviors.

Some additional behavioral indicators I suggest should be considered are; the place where the fact is perpetrated (according to Devinsky et al., 2009), and the frequency of sexual abuse during infancy in acquired pedophiles.

Also, gender is a variable to investigate, maybe the frequency of acquired pedophilia is higher in female than those of developmental one.

- Agree with these results.
- Acquired and developmental pedophilia are distinct entities, I do agree with this hypothesis. While both behavioural outcomes fall under the umbrella of paraphilia, acquired pedophilia appears to be the results of disruptive biological factors. However, it is important to nuance some elements. Not all rape or sexual assault commit on children are done by pedophile. I do remember reading a study mentioning some of the preparators of children sexual assaults weren’t paedophiles (a minor happens to be the victim, but adults were their interest, simply no adult were contextually “available” at the time). Accordingly, I suspect acquired pedophile may not necessarily have to target children to satisfy their sexual urges (in contrasst with developmental pedophile). Some acquired pedophilia may be determined by contextual situation.
- the behavioral indicators may widely differ in different defendants. A constant is certainly impulsive behavior, action not planned. what really make the difference is the temporal fracture for the pedophilic behavior insurgence. I do not believe that age can be a reliable indicator as while age can identify individuals with dementia, tumors and strokes can happen at every age.
- In general, I do agree with these results. However, I also believe that, if more cases would be available, two different patterns would emerge: first, a defendant might have an irresistible impulse and be able to understand the moral disvalue of the behavior. in this first case, the defendant is characterized by impulse dis control. second, a defendant might have control over his behavior, but might have an impaired ability to understand the moral disvalue of his action. in this second case, the behavior is done as the defendant do not recognize the behavior as wrong, despite being able to control it. Additional behavioral indicators might be searched in daily life habits and outside the modus operandi. For instance, absence of premetitation is indicative of dis.inhibition: is this dis.inibition also present in other aspects of the defendant life? etc.
- Although there may be on average differences regarding premeditation, masking, confession and guilt between acquired pedophilia and developmental pedophilia, one must be wary of a strict demarcation between individuals belonging to either group. Individuals with developmental pedophilia may also experience a sense of guilt for example, depending on the individual in question.
- Since acquired pedophiles behave in most cases with an impulse discontrol or an insuficet moral judgment their actions are guided by occasional event in these cases their actions shoould not be violent nor aggressive but just as if they are conducting a normal behavior. So a lack of phisical violence or cohercion while committing the offense could be another behavioral charactheristic of acquired pedophilles, while developmental pedophiles if contrasted in their plans to offend might exert violence as an interfearence to their goal oriented behavior, planned and premeditated in detail.

**Question n.2 - Neuroscientific investigation**

In our recent paper, we identified a possible behavioral profile of acquired pedophilia but we did not suggest any indication to support clinicians in the diagnostic process. In your opinion, how would it be possible to identify an acquired pedophile in neurological terms? Do you want to suggest any diagnostic tool or medical exam that might be helpful in the identification process? Do you think that it is possible to simulate this condition in any of these exams? What do you think is important to take into consideration for the best identification of an acquired pedophile?

- For acquired pedophiles, the key issues are onset associated with a provocative event (eg, development of frontal lobe tumor, head trauma, encephalitis, etc), change in patient's previous behavior that is temporally related. In cases where there is a relatively acute/subacute behavioral change without a known cause, the differentiation is more difficult.
- I think it's the before and after, in that what was their behavior like before a brain injury, tumor, etc. If there was no indication of pedophilic interests and "normal" sexual behavior, that is the main indicator of acquired pedophilia.
- Acquisition would be indicated by the longstanding lack of a previous history. Neuropsychological tests of impulse control, such as the Iowa Gambling Task, could prove informative. Neurologic signs of frontal lobe dysfunction could also prove informative.
- A clear evidence of a cerebral lesion in cerebral areas that can be connected with sexual or moral behavior. An occipital lesion, p.e, would be irrelevant.
- Neuropsychological examination and neuroimaging.
- Yes, I agree, it's possible to identify an acquired pedophile neurologically.

Due to its characteristics acquired pedophilia seems a disruption of the connections between various structures in the brain, so the various neuroimaging techniques are helpful, Diffusion Tensor Imaging (DTI) could be a tool that helps shed light to acquired pedophilia circuity.

Also, psychophysiological investigations are useful: heart rate, startle reflex and skin conductance could lead the forensic expert to a correct exclusion of psychopathic traits in acquired pedophiles.

Finally, hormonal and genetical aspect (PRGN & TAU) needs more attention in the scientific debate over acquired pedophilia.

- Neuroimaging (MRI, FDG-PET) may be helpful in order to identify focal brain abnormalities. Additionally genetic investigations may pave the way to discover neurobiological signatures of acquired pedophilia.
- Identifying acquired pedophilia should not be reduced to neurology. It is a multifactorial phenomenon; the large majority of patients with brain tumour will never develop paraphilic behaviours. I am not a MD, but I suspect a psychological tool should be developed to capture the subjective experience of acquired pedophilia; such instrument specifically designed to identify acquired pedophilie unique psychology (distinct from developmental) would help to come with a diagnostic.
- I do believe that, if pedophilia is of acquired origin, it does not come alone, but it should be coupled with other cognitive or neurological symptoms that might not be clinically related to pedophilia, but are anatomically related to it. I would suggest an in depth neuropsychological assessment particularly based on frontal functions assessment. As cognitive assessment can be partially simulated, particular attention should be paid to potential simulation and adeguate tests should be administered to catch it, if present. Furthermore, I would suggest a neurological examination and a structural MRI. these latter two exams are not possible to be malingered.
- As I have already expressed in my publications on the topic (see the one in Neuroetchics), acquired pedophilia is a symptom of a brain disorder. the brain disorders leading to pedophilia should also cause some other symptoms. Thus, clinicians should perform a neurological examination, psychiatric examination, they should also collect a detailed anamnesis in order to understand if additional symptoms (even if they are not legally relevant and thus not directly related to the pedohilic behavior) are present or not. An MRI is necessary to understand whether a brain based disorder is present or not. The brain disorder should be identified by naked eyes and no statistical analysis on MRI data should be performed. Finally, I would also suggest to perform an IAT to track back the temporal insurgence of the pedophilic urges. no-one of the above mentioned examinations might be malingered. finally, a neuropsychological battery to investigate cognitive symptoms(in particular on social cognition and impulse control) should be performed, with particular focus on malingering.
- In neurological terms: by using brain imaging that can detect relevant brain damage, tumors and/or neurological disorders. Brain imaging needs to be used in addition to behavioral diagnosis in order to make the diagnosis as reliable as possible.
- The possibility to mimic an acquired condition is always present and could open for resistance in the public prosecution, thus it is of paramount importance that all biological and psychological evidence is immune, as much as possible, from the simulation. MRI is impossible to simulate at a macroscopic level, especially if the areas affected by the lesion are already known to be involved in neural connection possibly producing pedophilia. But not only, since research in this topic is not sufficiently developed. High levels of Levo_dopa prescription have medical records, and the comorbidity of hypersexuality, impulse dyscontrol in other domains like gambling, can be objectively documented trough third parts. As a general rule in case of acquired pedophilia, the neurological damage should not produce only attraction to children but also other behavioral indicators, as comorbidity.

**Question n.3 -Neurological condition possibly leading to pedophilia**

The systematic review of literature identified some neurological disorders that in rare cases might lead to pedophilia: i.e. brain tumor, frontotemporal dementia, etc. Are there any other cases you know of? Are you aware of any other medical condition that can cause pedophilic behavior? Do you think the actual research on the topic is adequate or do you think there are new important issues to be taken into consideration? In your opinion, how important can the correct identification of acquired pedophilia be for the research on neurological and psychiatric disorders?

- Post- epilepsy surgery, encephalitis, autoimmune disorders, trauma. There is limited research both because of relative infrequency outside of dementia and the difficulty that both patients and physicians have in discussing or considering this.
- Parkinson's... the correct identification of acquired pedophilia for research on certain neurological and psychiatric disorders IS VERY IMPORTANT.
- I would also include TBI and encephalitis. Research into the topic seems reasonably adequate. The proper etiologic assignment is worthwhile as it may indicate a medical intervention, and could guide legal system interventions.
- It can be important, although is extremely rare. It would probably be more useful to concentrare in "abnormal sexual behaviors" in the listed conditions.
- Very important, mainly in addressing major psychiatric disorders at he onset of pedophilic behavior.
- As said before genetical aspects should be further investigated and also an epidemiological study is needed, the only one I know is (Henn et al., 1976).

Brain areas involved in acquired pedophilia seems Frontal lobe and Limbic system, if frontal areas are involved in impulsivity behaviors and sexual preference is influenced by subcortical structures, especially hypothalamus and amygdala, interactions between these structures should be investigated.

Are frontal areas involved in acquired pedophilia also involved in the integration between central and peripheric nervous signals? Could it be that acquired pedophilia is an impairment in this integration mechanism?

Finally the role of serotonin (and catecholamines in general) seems very central in acquired pedophilia. Serotonin network in acquired pedophiles should be clarified in order to assess the right medical treatment for this class of patients.

- I'm not aware of any additional neurological disorder linked to acquired pedophilia. The actual research on this topic is absolutely inadequate. Linking abnormal behavior to brain dysfunctions is paramopunt both for diagnosis and therapy.
- Nothing to contribute, aside from literature review I published.
- I am not aware of any condition, but I can imagine that every pathology of hypothalamus and OFC might potentially lead to acquired pedophilia. for instance , aneurysm of anterior communicant. The research is so far inadeguate.
- Potentially, any brain based disorder can lead to pedophilia. I am not aware of any other case, but a lesion in any area connected to the frontal lobe might cause pedophilia. Research on the topic is still inadeguate, questionnaire investigating sexual behaviors should be given to clinicians working with at risk population (neurologic). The correct discrimination between the two forms of pedophilia is of extreme relevance as it might advance the reseach in this under investigated topic. for instance, paraphilias in general are the only psychiatric disorders within the DSM5 that do not have the criteria saying that "the disorder should not be better explained by an underlying medical condition". Further evidence on this topic might help in improving diagnostic criteria within the DSM5 and, as consequence, the reliability of psychiatric diagnosis.
- Capsulotomy for OCD, and perhaps other forms of brain surgery, can cause clinical sexual disinhibition (based on a paper reporting on a number of patients), and thus potentially increase the risk of pedophilic transgressions. Deep brain stimulation for movement disorders may result in hyper-sexuality, impulse control disorders and disinhibition, which may increase the risk of pedophilic behavior. The correct identification of acquired pedophilia is important with respect to prevention (e.g. by removing the brain tumor and monitoring the patient post-op).
- Multi infartual or multi ischemic cortical insults in many cases due to chronic hypertension, being diffused and randomly distributed might occasionally produce an acquired pedophilia syndrome. In general, I would expect the higher frequency in Dementia, particularly fronto-temporal, secondarily tumors or lesions affecting deep structures such as the hypothalamus, and any cortical region temporal or orbitofrontal, thirdly biochemical imbalances due to Parkinson treatment, then occasionally other cases.

**Question n.4 - Consequences of misdiagnosis**

In our paper, we stated that the misdiagnosis of acquired pedophilia might have consequences both for the sexual offenders (some of the neurological disorders are life threatening conditions) and for the victims (being acquired pedophilia reversible in some cases, the correct identification might prevent further child abuses). Furthermore, there are ethical risks as well as it would be an ethical concern to put in jail someone who has a life threatening condition impacting on his behavior and who could benefit from a medical treatment. Do you agree/disagree with these opinions? If you disagree, can you explain why? In your opinion, what are the possible ethical and medical risks related to a missed identification of acquired pedophilia?

- I agree.
- I agree.
- I agree.
- This cuts both ways. Misdiagnosis is bad, but an acquired pedophile may unfortunately not be able to change their behavior - especially if there dementia or impaired frontal lobe functions. In such cases, jail is not ethical but the individual must be closely monitored to make sure children are not harmed in the future.
- I agree. The main risk of missing the diagnosis is missing a potentially treatable etiology.
- I strongly agree, with your consideration, missed identification could lead to social stigma in the offender and his family members.
- I totally agree with aforementioned statements.
- Ethical issues are centrals to diagnostic and treatment of acquired pedophilia. From a medical perspective, identifying an neurological origin to explain a disruptive sexual behaviours suggests we HAVE to do something to rectify the underlying medical issue. The ethical question come down to what we SHOULD do with individuals who fulfil the diagnostic of acquired pedophilia. It engages with our social constructions linked to a fair treatment of individual involved in deplorable sexual crime on children. The main risk is to impose an inappropriate legal solution on these individuals (retributive versus restorative option). It is difficult to see how a retributive sanction may be fair and provide justice given the presence of neurological disruptive causes. At the same time, it is hard to articulate a general prescription to all individual fulfilling the diagnostic of acquired pedophilia; a case by case solution seems more appropriate. Sending all acquired pedophile to medical treatment may result in converting some medical practices into punitive prescription in the long term.
- The major consequence is for the defendant's life. this is both a medical and ethical consequence.
- I do agree with these opinions. The consequences of misdiagnosis are potentially severe both for the defendant (that often suffer from life threatening conditions) and for his family (that is suffocated by social stigma). A better understanding of acquired pedoophilia is a medical and ethical need in the modern society.
- I fully agree. We have a responsibility to treat individuals with acquired pedophilia (right of offenders to receive medical care; and duty of society and the criminal justice system to prevent further victims).

At the same time, we also have this responsibility towards individuals with developmental pedophilia. It is important to correctly differentiate between individuals with acquired versus developmental pedophilia because these individuals will need different treatment and/or rehabilitation trajectories.

I have read the in press papers, and I strongly agree with many of the points. I would like to add the following to my previous response: It may be ethically problematic to provide a very stringent differentiation between individuals with acquired pedophilia and developmental pedophilia, at least in certain respects. I fully agree that neurologically the difference is apparent. However, when you write the following: "Thus, the modus operandi of developmental pedophiles is characterized by a highly predatory style, whereas the one used by acquired pedophiles seems to be not organized and it is characterized by an impulse discontrol." it is not fully clear to me what is meant. Are you only considering offenders with developmental pedophilia or all individuals with developmental pedophilia (including those individuals who did not commit any crimes)? I have learned from previous conversations with (forensic) psychiatric experts that they have several individuals with (developmental) pedophilia (who have not committed a crime to the best of their knowledge) visiting their clinic to ask for treatment on their own initiative because they are scared of sexually violating a child and do not want this to happen. I am not sure if all (or most) individuals with developmental pedophilia who are prone to commit a pedophilic offense or who have committed such an offense, are individuals with a highly predatory style, as is suggested in the paper. Perhaps more nuance and/or elaboration is necessary. Stigmatization of individuals with developmental pedophilia will not result in less crime, on the contrary. (see e.g. <https://www.hannapickard.com/responsibility-without-blame.html>).

- If a diagnosis of acquired pedophilia is established the offender is not a criminal, but is insane, but still, it is socially dangerous, hence he should undergo all possible reparatory medical intervention to restore situation 'quo ante' if possible, if not possible should be assigned to a non-reclusion but a condition where it's social danger is neutralized until recovered, if ever. Pedophiles in jail suffer extreme violence by other inmates usually, these offenders are not criminal and this is the message that should arrive at the families of these subjects. One thing is to realize that your relative was an undisclosed child predator, completely another to realize that your relative is a patient affected with an infirmity producing involuntarily inappropriate behavior. This aspect is now clear in Alzheimer patients, why shouldn't be for other neurological insults that produce inappropriate behavior?

**Question n.5 - Legal Consequences**

There is an ongoing debate on the legal consequences of acquired pedophilia. Do you believe that the identification of acquired pedophilia should influence the legal process and/or have repercussions on the legal consequences in the forensic field, especially of imputability? In your opinion, should the criminal charges be different in cases of acquired and developmental pedophilia?

- Yes, the charges and consequences should be different.
- Yes, the treatment associated with the individuals should be different, but the criminal charges may be the same.
- I believe they should be different and can be handled differently if there is a clear medical explanation for the symptom.
- The criminal charges can't be modified by medical or psychological opinions. The degree of guilty can me diminished by expert opinion. For my opinion an acquired form of paedophilia can be evaluated as insane.
- Of course.
- I strongly believe that acquired pedophiles shouldn't be legally prosecuted. In my opinion, criminal charges should be different for acquired pedophiles; medical interventions and pharmacological treatments are the gold standards for these offenders.
- In my opinion studying the neurobiological basis of acquired pedophilia has no direct consequence in the forensic field. In the future, new structures (hospices?) may be evaluated for these subjects.
- I do believe (I insist, it is a case by case reality -no systematic or automatic legal procedure) fulfilling the diagnostic of acquired pedophilia suggests a legal approach which is not inspired by retribution. A medical response may be more appropriate than a carceral one.
- The legal consequences might differs between acquired and developmental pedophilia. A clinician should evaluate whether or not the ability to resist to impulses are impaired or not. In general, acquired pedophilia are potentially to be considered not guilty by reason of insanity.
- I do strongly believed that the legal consequences on imputability should be widely different between developmental and acquired pedophilia. Developmental pedophilia are criminally liable. on the contrary, acquired pedophilia might be considered not guilty by reason of insanity. ALTHOUGH a case by case approach should be always indicated, I do believe that individual with acquired pedophilia are usually severity impaired in impulse dis control AND /OR moral reasoning. In any case, insanity should not be given only on the basis of the presence of a neurological disorder, but an impact of the neurological disorder on relevant behavior (moral reasoning and impulse control) should be assessed.
- Individuals with acquired pedophilia should receive treatment and not punishment. However, it is important to start a restorative justice trajectory if the victim(s) is/are willing to start such a process and could benefit from it - both in cases of acquired and developmental pedophilia.

My philosophical stance is such that moral guilt does not belong in criminal law and that retributive punishment is not defensible. Therefore, I do not think that criminal sanctions should be different in cases of acquired and developmental pedophilia. In both cases, retributive punishment is not defensible and not effective in terms of recidivism reduction. Rehabilitation and/or treatment and restorative justice processes are needed in both cases. If necessary for the safety of society and to prevent further victims, (temporary) civil commitment can be considered (based upon a clinical report) as a last resort.

It is however very likely that the wider society will find it easier to accept that an individual with acquired pedophilia receives treatment and not punishment in comparison to an individual with developmental pedophilia.

I would like to add the following to my previous answer: In the current guilt-based legal system, there are strong reasons to argue for differentiation in terms of criminal charges and legal consequences (treatment is an effective means for recidivism reduction in case of acquired pedophilia, whereas the situation in case of developmental pedophilia is more complex and treatment is less effective). However, we need to be wary of stigmatization towards individuals with developmental pedophilia when arguing for differential legal charges/consequences for individuals with acquired versus developmental pedophilia. We need to avoid 'populist' statements such as: 'individuals with a brain impairment couldn't help themselves whereas individuals with developmental pedophilia are monsters..." Lay people may tend to make such erroneous conclusions when learning about differential criminal charges based on neurological differences between these groups. All complex human behavior is the result of biopsychosocial factors in interaction, this is as much the case for behavior in the context of acquired pedophilia as it is the case in the context of developmental pedophilia.

- Acquired pedophilia diagnosis is not synonymous with insanity, the modus operandi of the offender supplement objective information of impulse control disorder of moral disinhibition. Acquired pedophilia must be an objective condition, and this will be only if further research on the brain areas involved in age- recognition, sex recognition and theory of mind in understanding the sexual availability of the partner, are well identified. An issue that now is not the case. Understanding that a physical lesion on a brain area can produce inappropriate sexual behavior independently of the free will of the offender should be progressively acquired as a fact by judges and criminal justice professionals. Hence yes in clear cases of acquired pedophilia with all or most of the behavioral indicators and the underlying neurological insult should lead to non-imputability due to insanity, and lack of free will. Acquired pedophile should not be condemned, but treated. A condemned any jail restriction is inadequate and useless for them that are patient and not criminals. Jail would be an unwanted stigma for the patient and the family. The family, on the contrary, should be supported and should be involved as possible to rehabilitate the offender.

**Question n.6 - Prejudice**

When we submitted our manuscript for publication, we found editors extremely reticent to publish the manuscript and many of them expressed concerns about publishing on the hypothesis of neurological insult causing pedophilic behavior. Do you believe there is any prejudice on this topic? If so, can you explain and give us some suggestions on how to diminish or remove this prejudice?

- Yes, our world is strongly constrained by political correctness, including science.
- There is a ton of prejudice, and it'll be hard to combat. All we can do is try to educate in order to better mitigate discrimination.
- I can't say that there is prejudice on this subject at journals.
- There is. Paedophilia is the last taboo in a otherwise sexual free society. I don't think there is a clear way to change this. Explaining mean also justification and the moral wall against paedophilia is very very high.
- Better explain the brain disorder related to the topic.
- I strongly believe that there is prejudice on this topic, sexually abusive behavior is one of the last topics mantled with prejudice. Now I don't have any suggestions in order to diminish this prejudice.
- Yes, publishing in this field is really difficult. We need more science on this topic.
- I have experienced substantial obstacles while trying to submit some of my work.

Formulating and publishing an international position paper will certainly help moving the debate further, at least it should contribute to diminish some of the academic ignorance.

- I do agree that there is a prejudice. the only way is to keep publishing and disseminating the results od research.
- The prejudice is huge, not only in lay people but also by the scientific community. I think it is extremely difficult to overcome this prejudice. I think that the only strategy consist in an honest dissemination of results: acquired pedophiles are criminals that cannot control their behavior. they are people suffering with a life threatening or other neurological condition that they cannot control. they are patients and not only defendants.
- There is prejudice about the neurological basis of behavior, including the hypothesis that neurological insult may cause pedophilic behavior. Prejudice arises (in part) due to ignorance regarding the causes of human behavior. Individuals (including many academics) need to be educated about the causes of human behavior (and evolutionary biology) and the multi-factorial basis (genes, brain, psychology, environment, and its interactions) of all complex human behavior (including moral and immoral behavior). This is a knowledge deficit that starts early (quality and content of education in schools) and it is difficult to remedy. Scientists have an obligation to inform the public about their research and to share their knowledge to the best of their ability in the public sphere.
- Yes, there is a prejudice, we are in an era of political correctness, everyone should behave as a standard response, there are issues that belong more to the Media than to science, and child offense is one of them. Media do not process, do not hear defense position, but act as an inquisition, accuse and judge without contradictory. Science should never be like this, should never be influenced by prejudice, however, due to political correctness assumption certain things cannot be expressed, among these to support or even instill the idea that an adult that sexually offends a child is not a monster. All the research on acquired pedophilia is seen as offering a hook to monsters to escape the right punishment. The only way to eliminate this is to do what has been done with Alzheimer's disease where the description of the behavioral abnormality of these patients went trough in the family, in the community in the media. If you have Alzheimer's disease you are allowed to indulge in inappropriate behavior some time very inappropriate behavior.... but among this inappropriate behavior for some unscientific reason, general people believe that child offense cannot be included. On the contrary child sexual offense can arise as to any other inappropriate behavior, is just our role to diffuse the results we achieved on acquired pedophilia.

**Question n. 7 - Additional thoughts**

Is there anything else you would like to add on this topic that could be helpful for the present consensus conference? Please remember that this is a generative phase, anything you write might be important and helpful for this project.

- No.
- May be worth trying to systematically survey a consecutive series of individuals charged or convicted with pedophilic activities in a defined region (eg, county or state) to better understand the frequency of both developmental and acquired pedophilia.
- This is a great project you are doing, I am very excited about this!
- No.
- Multicentric studies on abnormally sexual behaviors should be organized in selected populations, e.g. Hungtington, Dementia etc.
- Integrate the topic in a evolutionary frame.
- Just one consideration does the brain network involved in acquired pedophilia overlap, although partially, with the networks of other "paraphilias" (i. e. paraphilic coercive disorder and hypersexual disorder)?
- Nothing to add the moment.
- No.
- No other suggestions.
- Always include the perspective of victims in any paper concerning criminal justice responses to acquired and developmental pedophilia. Too often, the perspective of victims is not addressed. Victims often have a need to understand why an individual committed a particular crime. This project and the knowledge it generates is therefore very relevant with regard to the victims of pedophilic crimes. Perhaps if editors would consider this, it might be 'easier' for them to consider your work for publication (i.e. diminish their prejudice or fear).
- I think that we should work more also on the victim consequences on the offense by an acquired pedophile versus those of a developmental one. Finally, it is still an open issue to understand also in developmental pedophiles the origin of their behavior traumatic, cumulative, genetic, developmental and understand if all these offenders are criminals or in different ways insane and unable to control their behavior.
