# Supplementary material for: Acquired Pedophilia: international Delphi-method-based consensus guidelines
Source: Transl Psychiatry. 2023 Jan 18;13:11. doi: 10.1038/s41398-023-02314-8 (PMC9849353; doi:10.1038/s41398-023-02314-8)
Supplement: Supplementary file 5 — Supplementary Material E [file 41398_2023_2314_MOESM5_ESM.docx]

**Online form for the first Round of the Delphi Panel**

**Consensus Conference on Acquired Pedophilia**

- Which is your background? (please specify) (i.e. Neurologist, psychiatrist, neuropsychologist, ethics, forensic expert, medical sexologist, other)
- Before receiving the invitation to participate in this working group, were you aware of the existence of “acquired pedophilia”? YES/NO
- Do you believe that “pedophilia” can be a symptom of an underlying medical condition? YES/NO
- Do you know the difference between "acquired" and "developmental" pedophilia? YES/NO

If you want to take part in a consensus conference on this topic, please click YES and you will be redirected you to a questionnaire where you’ll be asked to rate your agreement on some statements. In this case, you will also be asked to provide your name to be included in the working group and in the eventual publication. This will be optional. If you don’t want to take part in this consensus conference, please click NO and the form will close.

***********************************

In the following form you will find some statements created based on the answers that you and other experts have given to a first preliminary questionnaire. You will be asked to rate your agreement on each statement on a likert scale (from 1= strongly disagree to 5 = strongly agree).

It will take approximately 15 minutes to reply.

If you don't know what to answer to one or more questions, please choose "3" (neutral/I don't know response).

***********************************

**BHAVIOURAL INDICATORS**

1. HOW MUCH DO YOU AGREE WITH THE STATEMENT: “Acquired and developmental pedophilia are two widely different disorders/distinct entities.”
2. HOW MUCH DO YOU AGREE WITH THE STATEMENT: “Acquired pedophilia occurs “de novo”, i.e. in individuals who have never manifested pedophilic interests or urges before, determining a behavioral fracture.”
3. HOW MUCH DO YOU AGREE WITH THE STATEMENT: “Unlike developmental pedophiles, acquired pedophiles behave in most cases with an impulse dis-control.”
4. HOW MUCH DO YOU AGREE WITH THE STATEMENT: “The impulse dis-control typical of individuals suffering from acquired pedophilia might manifest as lack of premeditation, un-planned actions and absence of masking the sexual abuse.”
5. HOW MUCH DO YOU AGREE WITH THE STATEMENT: “The impulse dis-control typical of individuals suffering from acquired pedophilia might have an impact on the physical places where abuses take place, as acquired pedophiles are likely to act in open or crowded spaces, where they can easily be seen.”
6. HOW MUCH DO YOU AGREE WITH THE STATEMENT: “The presence of premeditation is an important behavioral indicator that discriminates between an acquired and a developmental pedophile, especially when premeditation manifests in grooming behaviors.”
7. HOW MUCH DO YOU AGREE WITH THE STATEMENT: “Unlike individuals with developmental pedophilia, individuals with acquired pedophilia do not manifest a predatory behavior (i.e. active search for victims) but they are guided by occasional events.”
8. HOW MUCH DO YOU AGREE WITH THE STATEMENT: “Unlike individuals with developmental pedophilia, individuals with acquired pedophilia show a lack of rumination (i.e. obsession, constant thought about their victims, etc)."
9. HOW MUCH DO YOU AGREE WITH THE STATEMENT: “Acquired pedophilia can be characterized by the non-selective choice of the sexual partner OR it can be associated with general hypersexuality.”
10. HOW MUCH DO YOU AGREE WITH THE STATEMENT: “Unlike individuals with developmental pedophilia, individuals with acquired pedophilia do not usually show aggressive behavior during their abuses (physical violence other than the sexual one, coercion).”
11. HOW MUCH DO YOU AGREE WITH THE STATEMENT: “Unlike developmental pedophiles, acquired pedophiles behave in most cases with an insufficient moral judgment, which prevents them from understanding the wrongness of their action.”
12. HOW MUCH DO YOU AGREE WITH THE STATEMENT: “The insufficient moral judgment typical of individuals suffering from acquired pedophilia, might manifest as spontaneous confession or lack of sense of guilt, as they do not understand the moral and legal implications of their actions.”
13. HOW MUCH DO YOU AGREE WITH THE STATEMENT: “The impulse dis-control and the moral judgement deficits might be dissociated in acquired pedophilia: acquired pedophiles might act only as a consequence of an irresistible impulse although they are aware of the moral, social and legal disvalue of their acts OR they are only unable to understand that their behavior is morally, socially and legally wrong.”
14. HOW MUCH DO YOU AGREE WITH THE STATEMENT: “As acquired pedophilia occurs as a symptom of a neurological insult, it does not share with developmental pedophilia the psychological risk factors, as for instance having have been abused during infancy or high comorbidity with other psychiatric disorders.”
15. HOW MUCH DO YOU AGREE WITH THE STATEMENT: “While developmental pedophilia is usually present since adolescence, the age of the onset of acquired pedophilia is usually delayed due to its acquired origin.”
16. HOW MUCH DO YOU AGREE WITH THE STATEMENT: “To evaluate the presence of acquired pedophilia, additional behavioral indicators or abnormalities, indicative of impulse dis-control or of impaired moral judgment, might be present in daily life habits and outside the modus operandi.”
17. HOW MUCH DO YOU AGREE WITH THE STATEMENT: “A detailed anamnesis should be carried out in order to understand whether or not additional behavioral indicators (not legally relevant and present in the individual's daily life) are present.”
18. HOW MUCH DO YOU AGREE WITH THE STATEMENT: “Unlike developmental pedophiles, acquired pedophiles are likely to present concomitant focal cognitive alterations, as a consequence of the brain disorder that also causes acquired pedophilia.”
19. HOW MUCH DO YOU AGREE WITH THE STATEMENT: “Unlike developmental pedophiles, acquired pedophiles are likely to present concomitant neurological symptoms and signs indicative of brain sufferance.”
20. HOW MUCH DO YOU AGREE WITH THE STATEMENT: “Acquired pedophilia is a multifactorial phenomenon and for this reason a psychological tool should be developed to capture the subjective experience of acquired pedophilia.”

**NEUROSCIENTIFIC INVESTIGATION**

1. HOW MUCH DO YOU AGREE WITH THE STATEMENT: “If acquired pedophilia is suspected, an in-depth neuro-scientific investigation is warranted to further explore this condition.”
2. HOW MUCH DO YOU AGREE WITH THE STATEMENT: “Acquired pedophilia cannot be the sole symptoms of an underlying brain insult, but additional behavioral alteration, neurological symptoms, etc., should be present as well.”
3. HOW MUCH DO YOU AGREE WITH THE STATEMENT: “The majority of neuro-scientific evidences (i.e. brain scans, neurologic symptoms) cannot be malingered.“
4. HOW MUCH DO YOU AGREE WITH THE STATEMENT: “Including a NEUROPSYCHOLOGICAL EXAMINATION within the neuro-scientific investigation, it can be helpful to discriminate between developmental and acquired pedophilia as individuals with acquired pedophilia might present neuropsychological deficits consistent with the underlying neurologic disorder.”
5. HOW MUCH DO YOU AGREE WITH THE STATEMENT: “Neuropsychological tests measuring the ability to control impulses (such as the go/no go task, etc.) can be useful to investigate whether the impulse control component is spared or impaired. If the impulse component is impaired even in a neutral task, acquired origin of pedophilia might be suspected.”
6. HOW MUCH DO YOU AGREE WITH THE STATEMENT: “Neuropsychological tests measuring moral judgement (such as the ability to discriminate right from wrong, to identify a normal behavior, to evaluate the severity of a behavioral violation, etc) can be useful to investigate whether the moral component is spared or impaired. If the moral component is impaired, even in a neutral task, acquired origin of pedophilia might be suspected.”
7. HOW MUCH DO YOU AGREE WITH THE STATEMENT: “Neuropsychological impairment can potentially be malingered. Thus, particular attention should be paid to this. Neuropsychological tests accounting for malingering of impulse discontrol and impairment in moral judgement should be developed and used.”
8. HOW MUCH DO YOU AGREE WITH THE STATEMENT: “Including a PSYCHIATRIC ASSESSMENT within the neuro-scientific investigation it can be helpful to discriminate between developmental and acquired pedophilia as acquired pedophiles are not expected to show high comorbidities with other psychiatric disorders (in particular with personality disorders), differently from developmental pedophiles.”
9. HOW MUCH DO YOU AGREE WITH THE STATEMENT: “Including a NEUROLOGICAL ASSESSMENT within the neuro-scientific investigation it can be helpful to discriminate between developmental and acquired pedophilia, as acquired pedophilia might be associated with signs and symptoms typical of the underlying neurological disorder.”
10. HOW MUCH DO YOU AGREE WITH THE STATEMENT: “Neurologic signs of frontal lobe dysfunction can be a key characteristic of acquired pedophilia.”
11. HOW MUCH DO YOU AGREE WITH THE STATEMENT: “Including NEUROIMAGING within the neuro-scientific investigation it can be helpful to discriminate between developmental and acquired pedophilia as acquired pedophilia should be originated by a neurological disorder usually visible at brain scan (unless in its very early stage).”
12. HOW MUCH DO YOU AGREE WITH THE STATEMENT: “Brain imaging should be coupled with behavioral diagnosis in order to make the diagnosis of acquired pedophilia as reliable as possible.”
13. HOW MUCH DO YOU AGREE WITH THE STATEMENT: “Brain insult leading to acquired pedophilia should be clearly evident. In other words, subtle brain abnormalities emerging only after a statistical analysis of the brain scans (for instance using Voxel Based Morphometry, etc) could not be used as evidences supporting the presence of acquired pedophilia. Indeed, psychiatric disorders (i.e. developmental pedophilia) might be characterized by subtle abnormalities as well.”
14. HOW MUCH DO YOU AGREE WITH THE STATEMENT: “The brain pathology associated with the behavioral variant of fronto-temporal dementia (bvFTD) might not be clearly evident in the early stage of the disorder, making bvFTD an exception to the previous statement. However, the fast progression of the disorder and the possibility to support its diagnosis using alternative brain imaging methods (i.e. PET) can help in the diagnostic differentiation.”
15. HOW MUCH DO YOU AGREE WITH THE STATEMENT: “Including PSYCHOPHYSIOLOGICAL INVESTIGATIONS, like heart rate, startle reflex and skin conductance, within the neuro-scientific examination can be helpful to determine the presence of acquired pedophilia as these techniques can be helpful to exclude psychopathic traits.”
16. HOW MUCH DO YOU AGREE WITH THE STATEMENT: “Hormonal or biological analysis might support the possible presence of acquired pedophilia. Genetic investigations (PRGN & TAU) reveal neurobiological risk factors of acquired pedophilia.”
17. HOW MUCH DO YOU AGREE WITH THE STATEMENT: “The IMPLICIT ASSOCIATION TEST (a behavioral test based on the compatibility effect and the analysis of reaction times) could help to support the late onset of pedophilic urges.”
18. HOW MUCH DO YOU AGREE WITH THE STATEMENT: “Questionnaires investigating sexual behaviors should be provided to clinicians working with at risk populations (for example to all patients with specific neurologic disorders like dementias, Hungtington’s disorder, Parkinson’s disorder etc.) to further explore the possible insurgence of pedophilic tendencies in these patients.”

**NEUROLOGICAL CONDITION**

1. HOW MUCH DO YOU AGREE WITH THE STATEMENT: “Acquired pedophilia emerges as a symptom of brain disorder. Despite acquired pedophilia has been described following brain tumor, traumatic injuries, surgical lesions, encephalitis, multiple sclerosis, dementias, etc, theoretically, pedophilia can occurs as a symptom of any brain disorder.”
2. HOW MUCH DO YOU AGREE WITH THE STATEMENT: “Regardless the specific aetiology of the underlying neurological insult, it is of the utmost importance to determine a strong temporal link between the onset of the neurological insult and the insurgence of the pedophilic tendencies.”
3. HOW MUCH DO YOU AGREE WITH THE STATEMENT: “The brain network involved in pedophilia is still unknown and need to be further investigated. Of note, the relevance of connections/dysconnections to the frontal lobe should be specifically assessed.”
4. HOW MUCH DO YOU AGREE WITH THE STATEMENT: “Despite the brain network involved in acquired pedophilia is still unknown, any lesion affecting hypothalamus can potentially cause acquired pedophilia, as some nuclei of the hypothalamus are relevant for sexual orientation.”
5. HOW MUCH DO YOU AGREE WITH THE STATEMENT: “Despite the brain network involved in acquired pedophilia is still unknown, any lesion affecting the limbic system can potentially cause acquired pedophilia, as the limbic system is relevant for sexual behaviors and emotions.”
6. HOW MUCH DO YOU AGREE WITH THE STATEMENT: “Despite the brain network involved in pedophilia is still unknown, any lesion affecting orbitofrontal cortex can potentially cause acquired pedophilia, as the orbitofrontal cortex is relevant for impulse control.”
7. HOW MUCH DO YOU AGREE WITH THE STATEMENT: “Despite the brain network involved in pedophilia is still unknown, any lesion affecting ventro-medial and/or dorso lateral pre frontal cortex can potentially cause acquired pedophilia, as these brain regions are relevant for moral judgement.”
8. HOW MUCH DO YOU AGREE WITH THE STATEMENT: “Besides disorders with specific aetiology, acquired pedophilia can also emerge due to biochemical imbalance (e.g. addiction to dopaminergic drugs to treat Parkinson’s disease).”
9. HOW MUCH DO YOU AGREE WITH THE STATEMENT: “Deep brain stimulation for movement disorders may result in hyper-sexuality, impulse control disorders and disinhibition, which may increase the risk of pedophilic behavior.”
10. HOW MUCH DO YOU AGREE WITH THE STATEMENT: “Epidemiological studies are needed to be able to better evaluate the prevalence and incidence of acquired pedophilia.”
11. HOW MUCH DO YOU AGREE WITH THE STATEMENT: “To date, pedophilia and other paraphilias are the only psychiatric disorders within the DSM 5 that do not include the diagnostic criteria: <<the symptoms are not attributable to the physiological effects of a substance or another medical conditions>>. As acquired pedophilia is a clear entity, we suggest this criteria should be added to pedophilia within the next DSM edition."

**CONSEQUENCES OF MISDIAGNOSIS**

1. HOW MUCH DO YOU AGREE WITH THE STATEMENT: “It is important to differentiate developmental from acquired pedophilia as the two require different treatments and/or rehabilitation trajectories.”
2. HOW MUCH DO YOU AGREE WITH THE STATEMENT: “It is important to correctly differentiate between individuals with acquired versus developmental pedophilia, because acquired pedophiles often need medical treatment to both, treat the underlying neurological disease and to arrest the pedophilic tendency.”
3. HOW MUCH DO YOU AGREE WITH THE STATEMENT: “The misdiagnosis of acquired pedophilia might have drastic consequences for the sexual offender as acquired pedophilia often has a treatable aetiology. Thus, a misdiagnosis has an impact on the defendant’s health (and possibly on his life).”
4. HOW MUCH DO YOU AGREE WITH THE STATEMENT: “As acquired pedophilia often origins from a treatable underlying disorder and a "restitutio ad integrum" has been described following treatment of the underlying neurological condition, a correct diagnosis might help in preventing further sexual offenses/recidivism.”
5. HOW MUCH DO YOU AGREE WITH THE STATEMENT: “The consequences of the misdiagnosis of acquired pedophilia are potentially severe for the defendant’s family, that is suffocated by social stigma. The right diagnosis might help relatives to have a rational explanation of their relative’s behavior. Indeed, from the psychological point of view it is very different to consider a relative as sexual predator or as a patient with a neurological disorder producing involuntary inappropriate behavior.”
6. HOW MUCH DO YOU AGREE WITH THE STATEMENT: “The misdiagnosis of acquired pedophilia might potentially have ethical consequences, as it would be an ethical concern to put in jail someone who has a life-threatening condition impacting on his behavior and who would benefit more from medical treatment. In these cases, the offenders should be in any case closely monitored to avoid re-offending.”
7. HOW MUCH DO YOU AGREE WITH THE STATEMENT: “The misdiagnosis of acquired pedophilia might potentially have ethical consequences, as it would be unethical to impose an inappropriate legal solution based on a retributive penalty instead of a rehabilitative option."
8. HOW MUCH DO YOU AGREE WITH THE STATEMENT: “Attention should be paid to the risk of stigmatization of both, developmental and acquired pedophilia. As there are pedophiles who have not committed sexual crimes and who are actively asking for help, their stigmatization should be avoided, as it will not help in preventing crimes.”

**LEGAL CONSEQUENCES**

1. HOW MUCH DO YOU AGREE WITH THE STATEMENT: “A case by case approach is the most appropriate when establishing the legal consequences of acquired pedophilia.”
2. HOW MUCH DO YOU AGREE WITH THE STATEMENT: “The legal consequences on insanity should be different between developmental and acquired pedophilia."
3. HOW MUCH DO YOU AGREE WITH THE STATEMENT: “While defendants with developmental pedophilia are to be considered criminally liable, defendants presenting with acquired pedophilia can be potentially considered not guilty by reason of insanity.”
4. HOW MUCH DO YOU AGREE WITH THE STATEMENT: “When acquired pedophilia is suspected, the presence of an underlying neurological condition is not per se enough to lead to insanity, but the impact of the neurological condition on relevant behaviors (i.e. moral reasoning and impulse control) should be carefully assessed.”
5. HOW MUCH DO YOU AGREE WITH THE STATEMENT: “Acquired pedophiles should be assigned to a non-reclusive condition, however they should be treated in places where their social danger is neutralized until recovery.”
6. HOW MUCH DO YOU AGREE WITH THE STATEMENT: “Acquired pedophiles should not be condemned to jail, as jail restriction is inadequate as they need to be treated rather than/before being punished.”
7. HOW MUCH DO YOU AGREE WITH THE STATEMENT: “New structures (hospices or something else) may be evaluated for acquired pedophiles.”
8. HOW MUCH DO YOU AGREE WITH THE STATEMENT: “Determining whether the individual suffering from acquired pedophilia had pre-morbid sexual interests towards children but never acted them, is a scientifically interesting question. However, it is not legally important, as individuals should be judged from their behaviors."

**ISSUES AND FUTURE PERSPECTIVES**

1. HOW MUCH DO YOU AGREE WITH THE STATEMENT: “There is definitively a prejudice within the scientific community on this topic.”
2. HOW MUCH DO YOU AGREE WITH THE STATEMENT: “The existing prejudice on this topic will be very hard to overcome. Indeed, providing an explanation is considered equivalent to providing a justification.”
3. HOW MUCH DO YOU AGREE WITH THE STATEMENT: “The only way to diminish the prejudice on this topic is to publish and disseminate the results of research, to provide a better explanation of how the brain disorders can lead to acquired pedophilia, which are the associated behavioral indicators, and how can sexual offense be prevented in these patients.”
4. HOW MUCH DO YOU AGREE WITH THE STATEMENT: “The only way to overcome prejudice is to keep people informed with the latest scientific results: acquired pedophiles, despite they have committed a crime, are patients that need medical treatment.”
5. HOW MUCH DO YOU AGREE WITH THE STATEMENT: “It will be useful to include the perspective of victims in any paper concerning criminal justice responses to acquired and developmental pedophilia. Too often, the perspective of victims is not addressed. Victims often have a need to understand why an individual has committed a particular crime.”
6. HOW MUCH DO YOU AGREE WITH THE STATEMENT: “Neuropsychology teaches us that every cognitive component might be selectively damaged. Sexual behavior is a complex function that requires the integrity of many cognitive components (gender recognition, age estimation, moral reasoning, theory of mind, impulse control, to name a few) in order to be carried out. It is thus unreasonable to believe that sexual behavior, being the result of many complex cognitive tasks, could not be affected by neurological damage."
7. HOW MUCH DO YOU AGREE WITH THE STATEMENT: “Further studies should try to better estimate the prevalence and incidence of acquired pedophilia, by systematically surveying a consecutive series of individuals charged or convicted with pedophilic activities in a defined region (eg, county or state).”

**OTHER**

Do you have any additional thoughts or comments you would like to share with us?

Thank you for your participation.
