# Supplementary material for: Acquired Pedophilia: international Delphi-method-based consensus guidelines
Source: Transl Psychiatry. 2023 Jan 18;13:11. doi: 10.1038/s41398-023-02314-8 (PMC9849353; doi:10.1038/s41398-023-02314-8)
Supplement: Supplementary file 6 — Supplementary Material F [file 41398_2023_2314_MOESM6_ESM.doc]

**Online form for the second Round of the Delphi Panel**

**Email sent to colleagues:**

Dear Colleagues,

Thank you for expressing your agreement on the 76 statements!

We computed the percentage of agreement between the experts for each statement. The results are really interesting and will be shared with you very shortly. Before concluding this work, we would like to ask you to rate a second time a few statements (only 13) that did not reach the agreement at the first voting round. This second round is part of the Delphi method. As required by the method, we added a short explanation of the statement and /or proposal for a slight change. **It is very important for you to know that this second round has not been planned to convince you on the importance of voting each statement, but to understand why the consensus has not been reached** (i.e. was the statement wrongly written? Was the statement not clear?). So, feel free to vote according to your real opinion and feel free to not agree with the statements.

Many thanks for your important collaboration

Cristina Scarpazza & Andrea Camperio Ciani

**Introduction to the online form**:

In the following pages, you will be asked to rate again 13 statements. If you disagree, you will be asked to explain why (the answers could be very short as: I do not believe that XXX). In these open answers please feel free also to write that you are not sufficiently informed to agree on the statement, or something similar. If possible, please avoid to rate 3 (unsure) but try to express your opinion on agreement or disagreement.

**Online form:**

1 STATEMENT: “*Brain insult leading to acquired pedophilia should be clearly evident. In other words, subtle brain abnormalities emerging only after a statistical analysis of the brain scans (for instance using Voxel Based Morphometry, etc) could not be used as evidences supporting the presence of acquired pedophilia. Indeed, psychiatric disorders (i.e. developmental pedophilia) might be characterized by subtle abnormalities as well*”. **Agreement at the first round: 61.6%**

**Option: I agreed to this statement at the first round**

Our Concern: if this statement is not voted in agreement, our concern is that sophisticated neuroimaging analyses (e.g. Voxel based morphometry) could be asked in criminal trials and results highlighting subtle brain alterations might be used to support the presence of an acquired origin of pedophilia in every pedophiles. This would reduce the boundaries between developmental and acquired pedophilia with the potential for every pedophiles to be considered as acquired. Please note that this statement has a very high translational potential, i.e. please do not disagree with the statement because potentially or philosophically developmental pedophilia could have a neurologic/acquired unknown origin too (i.e. abnormal neuronal migration at birth).

After this explanation, how much do you agree with the above reported statement? (5 points likert scale)

If you disagree or you are unsure, could you please explain why?

2 STATEMENT: “*While defendants with developmental pedophilia are to be considered criminally liable, defendants presenting with acquired pedophilia can be considered not guilty by reason of insanity*”. **Agreement at the first round: 61.6%**

**Option: I agreed to this statement at the first round**

The statement “The legal consequences on insanity should be different between developmental and acquired pedophilia” reached 85% of agreement. Thus, it is not clear why we did not agree on the previous one. In particular, it is not clear whether you disagree with the first or the second part of the statement.

How much do you agree with the first part: “defendants with developmental pedophilia are to be considered criminally liable”? (5 points likert scale)

If you disagree or you are unsure, could you please explain why?

How much do you agree with the second part: “defendants presenting with acquired pedophilia can be considered not guilty by reason of insanity”? (please note that we used the term “can”. We are aware that the presence of pedophilia in individuals with brain insult might also NOT be of acquired origin. This statement wants to test if insanity is a relevant matter in individuals with pedophilia of suspected acquired origin) (5 points likert scale)

If you disagree or you are unsure, could you please explain why?

How much do you agree with the following reformulation: “It is relevant to assess insanity in individual with acquired pedophilia”? (5 points likert scale)

3 STATEMENT: “*The presence of premeditation is an important behavioral indicator that discriminates between an acquired and a developmental pedophile, especially when premeditation manifests in grooming behaviors*”. **Agreement at the first round: 69.3%**

**Option: I agreed to this statement at the first round**

How much do you agree with the first part of the statement: “The presence of premeditation is an important behavioral indicator that discriminates between an acquired and a developmental pedophile” (acquired pedophilia do not premeditate)? (5 points likert scale)

If you disagree or you are unsure, could you please explain why?

How much do you agree with the second part of the statement: “acquired pedophilia do not manifest grooming behaviors”? (5 points likert scale)

If you disagree or you are unsure, could you please explain why?

How much do you agree with the following reformulation?

“While premeditation is always present in developmental pedophilia, acquired pedophilies might act without premeditating the sexual assaults” (5 points likert scale)

4 STATEMENT: “*Unlike individuals with developmental pedophilia, individuals with acquired pedophilia show a lack of rumination (i.e. obsession, constant thought about their victims, etc)*”. **Agreement at the first round: 61.6%**

**Option: I agreed to this statement at the first round**

How much do you agree with the concept expressed in the first part of the statement: “individual with developmental pedophilia usually show rumination (i.e. obsession, constant thought about their victims, etc)”. (5 points likert scale)

If you disagree or you are unsure, could you please explain why?

How much do you agree with the concept expressed in the first part of the statement: “individuals with acquired pedophilia usually do not show rumination (i.e. obsession, constant thought about their victims, etc)”. (5 points likert scale)

If you disagree or you are unsure, could you please explain why?

5 STATEMENT: “*Unlike individuals with developmental pedophilia, acquired pedophilies behave in most cases with an insufficient moral judgement, which prevents them from understanding the wrongness of their action*”. **Agreement at the first round: 53.9%**

**Option: I agreed to this statement at the first round**

How much do you agree with the concept expressed in the first part of the statement: “individuals with developmental pedophilia have spared moral judgement: i.e. they are aware of the wrongness of their action”. (5 points likert scale)

If you disagree or you are unsure, could you please explain why?

How much do you agree with the concept expressed in the second part of the statement: “individuals with acquired pedophilia might have impaired moral judgement” (please note that we changed “in most cases” with “might have”)? (5 points likert scale)

If you disagree or you are unsure, could you please explain why?

How much do you agree with the concept expressed in the third part of the statement: “in the cases where moral judgement is impaired, individuals might not be aware of the wrongness of their actions” (5 points likert scale)

If you disagree or you are unsure, could you please explain why?

6 STATEMENT: “*The insufficient moral judgement typical of individuals suffering from acquired pedophilia might manifest as spontaneous confession or lack of sense of guilt, as they do not understand the moral and legal implications of their actions*”. **Agreement at the first round: 69.3%**

**Option: I agreed to this statement at the first round**

We propose to slightly modify the statement replacing the term “typical of” with the expression “that might be present in”.

How much do you agree with the concept expressed in the first part of the statement: “The insufficient moral judgement that might be present in individuals suffering from acquired pedophilia might manifest as spontaneous confession or lack of sense of guilt” (5 points likert scale)

If you disagree or you are unsure, could you please explain why?

How much do you agree with the concept expressed in the second part of the statement: “in the cases where moral judgement is impaired, individuals with acquired pedophilia do not understand the legal and moral disvalue of their actions” (5 points likert scale)

If you disagree or you are unsure, could you please explain why?

7 STATEMENT: “*Neuropsychological tests measuring moral judgement (such as the ability to discriminate right from wrong, to identify a normal behavior, to evaluate the severity of a behavioral violation, etc) can be useful to investigate whether the moral component is spared or impaired. If the moral component is impaired, even in a neutral task, acquired origin of pedophilia might be suspected*”. **Agreement at the first round: 69.3%**

**Option: I agreed to this statement at the first round**

Neuropsychological tests that measure moral judgement. For instance, one of them measure the ability of the individual to discriminate a normal from an abnormal behavior, i.e. Ralph wants to go to the park to run. Because it was a very warm day, Ralph decided to run naked. It is a normal or abnormal behavior? If it is abnormal, can you rate the severity of this behavior from 1 to 10?. Using this test, for example, neuropsychologists can identify individuals that show difficulty to discriminate normal from not normal behaviors or that underestimate the severity of behavioral violations.

Given this explanation, how much do you agree with the first part of the statement: “Neuropsychological tests measuring moral judgement (such as the ability to discriminate right from wrong, to identify a normal behavior, to evaluate the severity of a behavioral violation, etc) can be useful to investigate whether the moral component is spared or impaired.” (5 points likert scale)

If you disagree or you are unsure, could you please explain why?

Within the second part of the statement, for “neutral task” we meant a task not directly investigating the sexual component and their sexual urges towards children. We reasoned that if the moral judgement is impaired, this should be evident even in not-sexual tasks.

Given this explanation, how much do you agree with the second part of the statement: “If the moral component is impaired, even in tasks that do not investigate sexual behaviors, acquired origin of pedophilia might be suspected”.? (5 points likert scale)

If you disagree or you are unsure, could you please explain why?

8 STATEMENT: “*Including PSYCHIATRIC ASSESSMENT within the neuro-scientific investigation it can be helpful to discriminate between developmental and acquired pedophilia as acquired pedophiles are not expected to show high comorbidities with other psychiatric disorders (in particular with personality disorders), differently from developmental pedophiles*”. **Agreement at the first round: 69.3%**

**Option: I agreed to this statement at the first round**

If the second part of the statement would be removed (see example below), how much would you agree with it?

**“**Including PSYCHIATRIC ASSESSMENT within the neuro-scientific investigation it can be helpful to discriminate between developmental and acquired pedophilia” (5 points likert scale)

If you agree, could you please explain why is, in your opinion, psychiatric assessment important?

If you disagree, could you please explain why is, in your opinion, psychiatric assessment not important?

9 STATEMENT: “*The IMPLICIT ASSOCIATION TEST (a behavioral test based on the compatibility effect and the analysis of reaction times) could help to support the late onset of pedophilic urges*”. **Agreement at the first round: 61.6%**

**Option: I agreed to this statement at the first round**

We noted that many colleagues voted “3” at this item, while no-one clearly disagree. For this reason, we decided to provide you an explanation of the implicit association test (IAT) and its potential application (<https://implicit.harvard.edu/implicit/takeatest.html>).

The IAT is a behavioral task created to measure implicit associations. If you want to understand what an implicit association is, please try to think to drive a car with crossed legs: this would be very difficult as in your mind the right feet is used to press the accelerator, while if you cross the legs, the right feet would be used to press the clutch. In the similar way, the IAT can be used to test how strong is the association between different concepts or autobiographical events (if you want to read a review please see Agosta and Sartori, 2013: the autobiographical IAT: a review): for instance the association between woman and humanistic jobs vs man and scientific jobs, etc. In the case of acquired pedophilia, the autobiographical IAT might be created to test the association between true and false events (e.g. true event: “I am doing a test”; false event: “I am playing football”) with different versions of the same autobiographical event, for instance the insurgence of the pedophilic urges (e.g. recent insurgence: “only recently I feel to be sexually attracted by children”, vs past insurgence: “I have always been found attracted by children”).

According with the compatibility phenomenon, an individual would be much faster to respond to associated concept if they share the same motor pattern. In other words: if truth is associated with recent insurgence, then the individual will be faster to respond to true sentences and sentences describing a recent insurgence if he has to respond to both of them with the same hand. On the contrary, if this association exist, the individual will be slower if he is required to respond to these concepts with two different hands. It is also important to know that the result of this test cannot be malingered.

After this explanation, how much do you agree with the statement? (5 points likert scale)

If you disagree or you are unsure, could you please explain why?

10 STATEMENT: “*Acquired pedophilia emerge as a symptom of brain disorder. Despite acquired pedophilia has been described following brain tumor, traumatic injuries, surgical lesions, encephalitis, multiple sclerosis, dementias, etc, theoretically, pedophilia can occur as a symptom of any brain disorder*”. **Agreement at the first round: 61.6%**

**Option: I agreed to this statement at the first round**

Please note that this statement refers to different aetiologies of disorders possibly leading to acquired pedophilia and not lo lesion localization. We also realized that the statement sounds too strong if we say “any disorder”. If the statement would be changed replacing “any brain disorders” with “a large variety of brain disorder” and making clear we are referring to brain disorder with different aetiology (see the following reformulation of the statement), how much would you agree with it?

“Acquired pedophilia emerge as a symptom of brain disorder. Despite acquired pedophilia has been described following brain tumor, traumatic injuries, surgical lesions, encephalitis, multiple sclerosis, dementias, etc, theoretically, acquired pedophilia can potentially occur as a symptom of a large variety of neurological disorder with different aetiology”. (5 points likert scale)

If you disagree or you are unsure, could you please explain why?

11 STATEMENT: “*Despite the brain network involved in acquired pedophilia is still unknown, any lesion affecting hypothalamus can potentially cause acquired pedophilia, as some nuclei of the hypothalamus are relevant for sexual orientation*”. **Agreement at the first round: 53.9%**

**Option: I agreed to this statement at the first round**

That hypothalamus is relevant for sexual orientation has been repeatedly found in literature (see for instance: <https://www.ncbi.nlm.nih.gov/pubmed/1292983>; <https://www.pnas.org/content/105/30/10273;https://science.sciencemag.org/content/253/5023/1034>), so, we assume you do not disagree with this part of the statement. If we are wrong, please exlain in the comment part below.

If the statement would be changed replacing the term “any lesion” with “lesions” and replacing the term “cause” with “contribute or influence” (see the following reformulation of the statement), how much would you agree with it?

“Despite the brain network involved in acquired pedophilia is still unknown, lesions affecting the hypothalamus can potentially contribute to the insurgence (or: can potentially influence the insurgence) of acquired pedophilia, as some nuclei of the hypothalamus are relevant for sexual orientation.” (5 points likert scale)

If you disagree or you are unsure, could you please explain why? (DOMANDA APERTA)

12 STATEMENT: “*Despite the brain network involved in acquired pedophilia is still unknown, any lesion affecting the limbic system can potentially cause acquired pedophilia, as the limbic system is relevant for sexual behaviors and emotions*”. **Agreement at the first round: 69.3%**

**Option: I agreed to this statement at the first round**

That the limbic system is relevant for sexual behaviors and emotions is clear in the literature. If the statement would be changed replacing the term “any lesion” with “lesions” and replacing the term “cause” with “contribute or influence” (see the following reformulation of the statement), how much would you agree with it?

“Despite the brain network involved in acquired pedophilia is still unknown, lesions affecting the limbic system can potentially contribute to the insurgence (or: can potentially influence the insurgence) of acquired pedophilia, as the limbic system is relevant for sexual behaviors and emotions”. (5 points likert scale)

If you disagree or you are unsure, could you please explain why?

13 STATEMENT: “*Deep brain stimulation for movement disorders may result in hyper-sexuality, impulse control disorders and disinhibition, which may increase the risk of pedophilic behavior*”. **Agreement at the first round: 61.6%**

We reformulated the statement, replacing “and” with “or”. How much do you agree with the statement: **“**Deep brain stimulation for movement disorders may result in hyper-sexuality, impulse control disorders OR disinhibition, which may increase the risk of pedophilic behavior.”? (5 points likert scale)

If you disagree or you are unsure, could you please explain why?
