# Supplementary material for: Acquired Pedophilia: international Delphi-method-based consensus guidelines
Source: Transl Psychiatry. 2023 Jan 18;13:11. doi: 10.1038/s41398-023-02314-8 (PMC9849353; doi:10.1038/s41398-023-02314-8)
Supplement: Supplementary file 2 — Supplementary Material B [file 41398_2023_2314_MOESM2_ESM.docx]

**Invitation email:**

Dear Professor XXX,

We hope this email finds you well.

We are contacting you to ask whether you could be interested in writing an opinion on acquired pedophilia together with us. A month ago, we submitted an original investigation on acquired pedophilia whose abstract is attached at the end of this email. We would like now to write a review/opinion paper on acquired pedophilia aiming to provide the readers with clear guidelines on how to deal with patients with acquired pedophilia both from the clinical and forensic point of view. To this aim, we would like to create an international panel of experts.

As you are an international expert on the topic, we will be delighted if you can accept our invitation to join this international project. The interdisciplinary expert panel, led by Dr. Scarpazza (who will be the first author) and Prof. Camperio Ciani (who will be the last author), will include neurologists, psychiatrists, forensic neuropsychologists and experts in evolutionistic psychology and in neuro-ethic.

If you agree to actively take part to this project, please reply to this email. You will be provided with: the papers already published on the topic, a draft of our submitted paper and a bullet points draft describing how we would like to organize the opinion paper. Please feel free to suggest us additional experts in the field that might be interested in this collaboration.

We are looking forward to hearing from you

Many thanks and best wishes

Cristina Scarpazza & Adrea Camperio Ciani

********************************************************

**Follow up email:**

Dear Colleague,

a few months ago, you agreed to participate to a consensus conference aiming to identify consensus guidelines for the management of suspect cases of acquired pedophilia.

We are almost ready to start this exciting project. Before starting, we would like to share with you the existing literature on acquired pedophilia. Within this literature, we also included a recent paper published from our working group, where we identified a behavioral profile discriminating acquired from developmental pedophilia. We hope you will find it interesting.

In a week, you will receive an email where you will find a few open questions to understand your thoughts on acquired pedophilia. This step will give you the possibility to put your expertise and knowledge into the project. This first step will be followed by the Delphi panel.

Kind regards

Cristina Scarpazza & Andrea Camperio Ciani
